# Post-COVID-19 health inequalities: Estimates of the potential loss in the evolution of the health-related SDGs indicators

**DOI:** 10.1371/journal.pone.0305955

**Published:** 2024-07-24

**Authors:** Fabrício Silveira, Wanessa Miranda, Rômulo Paes de Sousa

**Affiliations:** 1 Oswaldo Cruz Foundation (FIOCRUZ), Belo Horizonte, Brazil; 2 Department of Health Management, Nursing School, Federal University of Minas Gerais, Belo Horizonte, Brazil; Imperial College London School of Public Health, UNITED KINGDOM OF GREAT BRITAIN AND NORTHERN IRELAND

## Abstract

This study delves into the global evolution of 43 Sustainable Development Goals (SDG) indicators, spanning 7 major health themes across 185 countries to evaluate the potential progress loss due to the COVID-19 pandemic. Both the cross-country and temporal variability of the dataset are employed to estimate an empirical model based on an extended version of the Preston curve, which links well-being to income levels and other key socioeconomic health determinants. The approach reveals significant global evolution trends operating in each SDG indicator assessed. We extrapolate the model yearly between 2020 and 2030 using the IMF’s pre-COVID-19 economic growth projections to show how each country in the dataset are expected to evolve in these health topics throughout the decade, assuming no other external shocks. The results of this baseline scenario are contrasted with a post-COVID-19 scenario, where most of the pandemic costs were already known. The study reveals that economic growth losses are, on average, estimated as 42% and 28% for low- and lower middle-income countries, and of 15% and 7% in high- and upper middle-income countries, respectively, according to the IMF’s projections. These disproportional figures are shown to exacerbate global health inequalities revealed by the curves. The expected progress loss in infectious diseases in low-income countries, for instance, is an average of 34%, against a mean of 6% in high-income countries. The theme of Infectious diseases is followed by injuries and violence; maternal and reproductive health; health systems coverage; and neonatal and infant health as those with worse performance. Low-income countries can expect an average progress loss of 16% across all health indicators assessed, whereas in high-income countries the estimated loss is as low as 3%. The disparity across countries is even more pronounced, with cases where the estimated progress loss is as high as nine times worse than the average loss of 8%. Conversely, countries with greater fiscal capacity are likely to fare much better under the circumstances, despite their worse death count, in many cases. Overall, these findings support the critical importance of integrating the fight against inequalities into the global development agendas.

## Introduction

The establishment of the Sustainable Development Goals (SDGs) in 2015 marked a significant triumph of globalism. The wide and integrated agenda, encompassing themes that range from eradicating poverty and promoting well-being to addressing socioeconomic inequalities and climate change, represent the most ambitious global development plan in history, built on notions of global partnership and solidarity. The initial optimism following its approval by the 193 UN member states, however, soon gave way to concerns as nationalistic ideals resurfaced worldwide. Moreover, the possibility of a pandemic and its broad disruptions were not even on the radar at that time. After a slow begin, where progress failed to meet the required speed and scale of change [1], the COVID-19 outbreak led to an unprecedented combination of health, economic, and humanitarian crises that resulted in further backlashes for several SDGs [2].

Despite uncertainties surrounding the extent to which the pandemic undermines progress and exacerbates challenges, experts concur that the confluence of crises imperils lives and livelihoods, rendering the attainment of targets even more arduous [1–3]. Indeed, the pandemic exposed vulnerabilities and tested global resilience, raising concerns whether the goals set in 2015 could still be attainable given the new challenges, even among the 2030 Agenda advocates [1, 3].

In fact, the long-term repercussions of the pandemic remain unknown [1]. Adding to the direct disease-related fatalities, the burden on health systems during critical care moments has further escalated avoidable deaths [4]. Presently, owing to limitations in health data systems, comprehensive information on the pandemic’s effects on population morbidity and mortality remains scarce. Nevertheless, emerging evidence suggests that the multifaceted crisis may have graver consequences than initially anticipated. Studies reveal a deteriorating outlook for noncommunicable diseases, evidenced by increasing sedentary lifestyles, reduced physical activity, increased alcohol and tobacco consumption, aggravated food insecurity, reduced utilization of health services and adoption of preventive measures, along with an increase in mental health disorders [5–7].

Furthermore, both the pandemic and measures implemented to mitigate its impacts have caused severe economic and social repercussions worldwide, disproportionately affecting marginalized communities, including women and other vulnerable groups. Job losses and diminished means of subsistence have hit current generations at an unprecedented scale. While skilled workers were able to transition to remote work, unskilled laborers predominantly engaged in informal sector activities faced reduced wages and heightened health risks. Furthermore, lockdowns and income losses have rendered health and education less accessible, particularly impacting the disadvantaged, including women and girls [8].

Moreover, the pandemic has amplified pre-existing disparities that have long constrained global development and constitute a significant focal point of the 2030 Agenda. Strikingly, the 2021 annual Sustainable Development Report marked the first decline in the rate of progress towards the SDGs since 2015, primarily attributable to the rise in poverty and unemployment rates following the onset of the COVID-19 pandemic [9]. It is worth noting that its final impact on the global SDG performance may be underestimated due to the lack of timely and comparable data.

Against this backdrop, the current study offers a fresh view of the “potential progress loss” attributable to the COVID-19 pandemic in 43 health-related indicators and 7 major health themes using counterfactual scenarios built for 185 countries in different income levels. Unlike the literature focusing on the pandemic impact on specific health topics and/or specific countries [5–8], this study provides a much broader, ecological, perspective of the pandemic’s repercussions on the potential evolution of health-related SDGs globally. At the same time, the study deepens the understanding of the dominating cross-country trends in each health topic and the factors explaining them, a differential, compared to global SDGs reports [1, 9]. Notably, the approach intends to measure the cost of the pandemic in terms of the potential evolution of the health phenomena measured by the SDG indicators, shedding light on the urgent need of a renewed global commitment to revert current trends and accelerate the health-related SDGs in the coming years, particularly in low-income countries, in the aftermath of the pandemic.

## Methodology

This study presents a static comparative assessment of the impact of changes in economic perspectives due to the COVID-19 pandemic on the potential evolution of 43 health-related Sustainable Development Goals (SDG) indicators. The empirical approach assumes the existence of transnational trends in health to estimate global counterfactual evolution curves for each indicator. These are extrapolated to show how each country in the dataset are expected to evolve in the health phenomena represented by each indicator (health topics) in the course of economic development, assuming no other external shocks, and how changes in economic expectations in a scenario post-COVID impact the initial forecasted trajectory of these indicators.

Data for this study were obtained from the official UN SDGs database [10]. The global and thematic coverage of the sample is illustrated in Table 1 below.

**Table 1:** Health-related themes and indicators: Data coverage.

Themes	Indicators		Coverage^1^		
			Countries	Initial year	Final year
Maternal and reproductive health	311	Maternal mortality rate (per 100,000 live births)	185	2000	2017
	312	Births assisted by qualified health personnel (%)	166	2000	2021
	223	Proportion of women aged 15–49 years with anaemia, pregnant (%)	192	2000	2019
	371	Childbearing-age women (15–49 years) with their need for family planning satisfied with modern methods (%)	86	2000	2020
	372	Adolescent birth rate (per 1,000 women 15 to 19 years)	193	2001	2020
Infant and neonatal health	221	Children <5 years with stunting (% height for age <-2 DP)	155	2000	2020
	222a	Children < 5 years with wasting (% weight/height <-2 DP)	92	2000	2020
	222b	Children <5 years with overweight (% weight/height >+2 DP)	93	2000	2020
	321	Mortality rate in children under 5 years (probability of dying by 5 years of age per 1,000 live births)	195	2000	2020
	322	Neonatal mortality rate (per 1,000 live births)	195	2000	2020
	3b1a	Immunization coverage for diphtheria, tetanus, and pertussis (DTP3) within 1 year	195	2000	2020
	3b1b	Vaccination coverage by country with second dose of vaccine including measles (MCV2)	179	2000	2020
	3b1c	Immunization coverage with conjugate pneumococcal vaccine (PCV3) in children under 1 year	147	2008	2020
Infectious diseases	331	New HIV infections (per 1,000 uninfected inhabitants)	132	2000	2020
	332	TB incidence (per 100,000 inhabitants per year)	215	2000	2020
	333	Malaria incidence (per 1,000 inhabitants at risk)	109	2000	2020
	334	Prevalence of hepatitis B surface antigen (HBsAg) in children under 5 years (%)	194	2015	2020
	335	Reported number of persons requiring interventions against neglected tropical diseases	194	2010	2019
Non-communicable diseases	211	Prevalence of undernourishment (%)	161	2001	2019
	341	Probability (%) of dying from 30 to 70 years of age of any cardiovascular disease, cancer, diabetes, or chronic respiratory disease	183	2000	2019
	342	Crude suicide rates (per 100,000 inhabitants)	183	2000	2019
	352	Total per capita alcohol consumption (recorded + unrecorded) (15+ years)	188	2000	2019
	3a1	Age-standardized prevalence of current smoking in persons 15 years or older	164	2000	2020
Violence and injuries	361	Death rate due to road traffic injuries (per 100,000 inhabitants)	183	2000	2019
	1311	Death rate from natural disasters (per 100,000 inhabitants)	155	2005	2020
	1611	Estimated homicide rates per 100,000 inhabitants	114	2000	2014
	1623	Proportion of population aged 18–29 years who experienced sexual violence by age 18, by sex (% of population aged 18–29)	46	2005	2020
Environmental risks	391	Mortality rate attributable to air and household pollution (per 100,000 inhabitants)	183	2016	2016
	392	Mortality rate attributed to exposure to unsafe WASH services (per 100,000 inhabitants)	183	2016	2016
	393	Mortality rate attributed to unintentional poisoning (per 100,000 inhabitants)	183	2000	2019
	611	Population that uses safely managed drinking water services (%)	136	2000	2020
	621a	Population that uses safely managed sanitation services (%)	122	2000	2020
	621b	Proportion of population practicing open defecation, by urban/rural (%)	222	2000	2020
	712	Proportion of the population using clean fuels and technologies	191	2000	2020
	1162	Fine particulate matter (PM2.5)	194	2011	2016
Health systems and coverage	1a2	Current health expenditure (CHE) as percentage of gross domestic product (GDP) (%)^2^	187	2000	2020
	381	Coverage of essential health services	183	2000	2019
	382	Proportion of population with large household expenditures on health (greater than 10%) as a share of total household income (%)	97	2000	2020
	3c1a	Physicians (per 10,000 inhabitants)	178	2000	2020
	3c1b	Nursing and obstetric personnel (per 10,000 inhabitants)	185	2000	2020
	3c1c	Dentists (per 10,000 inhabitants)	179	2000	2020
	3c1d	Pharmacists (per 10,000 inhabitants)	161	2000	2020
	3d1	Capacity of the IHR and health emergency preparedness (average of 13 points—SPAR)	193	2018	2020

Notes

^1^ Number of countries with at least one post-2015 data entry. The cross-sectional and longitudinal availability of data varies between indicators. The dictionary of indicators and complete information on each country´s longitudinal availability can be found in the official SDG global database.

^2^ Data series source: WHO.

Source: own elaboration with data from UNDesa. <https://unstats.un.org/sdgs/dataportal> Access in 19 April 2023.

To reconstruct the global evolution curves for each indicator, we employ a model based on the Preston curve, which links well-being (life expectancy) to income levels (per capita gross domestic product (*GDP*_pc_)) [11]. The empirical association between health and *GDP*_pc_ has been supported by several studies since the late 1970s [12–14]. These studies indicate diminishing returns to income in terms of life expectancy, where the marginal gains of health from GDP increase at lower income levels but decrease for higher income levels. Over time, the association between health and GDP has evolved, with some studies suggesting that new technologies have improved global health conditions regardless of a country’s economic performance [15]. More recent data, however, indicate a stronger relationship between health and GDP in the decades following Preston’s study [16].

To extend the original Preston curve model, we incorporate other key determinants of country health phenomena, such as overall health expenditures (% of GDP) from the World Health Organization (WHO) and the Gini index from the World Bank. The former proxies the availability and reach of health services, while the latter measures access to health services, as income inequalities often correlate with health inequalities within and across countries. The basic model is represented by Eq (1).

$${ln\;SDG}_{i,t}=\propto +\beta_{1}{ln{GDP}_{pc}}_{i,t}+\beta_{2}ln{Gini}_{i,t}+\beta_{3}ln{HE}_{i,t}+\beta_{4}{{\ln GDP}_{pc}}_{i,t}\times {R_{D}}_{i}+f_{i,t}+\varepsilon_{i,t}$$ (1)

where ∝ is a constant, β represents the fitting parameters, f and ε are the fixed effects and error term, respectively. Subscripts i and t represent countries and time, respectively. Natural logarithms are used for all variables, allowing the coefficients to be interpreted as the percentage rate of change in the SDG indicator due to a change in the explanatory variables.

We utilize both cross-country and time variance to fit the models, enabling the coefficients to reflect transnational trends and time drifts in the *GDP*_pc_ parameter. To reduce autocorrelation and capture long-term trends despite annual variance in the data, 5-year averages are used. This strategy also proved successful to generate a balanced panel for each health indicator. By adopting this approach and including temporal fixed effects, we address concerns raised about the original Preston curve, particularly regarding the lack of empirical evidence on the validity of the association over time in both serial and longitudinal studies.

The equation also incorporates an interaction between a Regional categorical variable (*R*_D_) and the *GDP*_pc_. This captures the influence of dynamic regional factors on the target health phenomena. The term exhibits a better fit compared with time-invariant regional dummies, especially with the introduction of individual fixed-effects. Additionally, we find that regional differences in the trajectories are significant for many indicators.

As envisaged by Preston, the preliminary analysis revealed important non-linearities in the evolution of some indicators (see Results section). This hypothesis is confirmed by the analysis of partial residuals for segments of the global curve and likelihood ratio tests applied to linear models and splines. The inclusion of multiplicative regional dummies improves the general fit of the estimations, partially reducing the error. However, since the error appears to be particularly correlated with the country development level, we estimate Eq 1 for subsamples of countries in four income groups, as defined by the World Bank in June 2023: (i) low-income countries, with per capita GDP of $1,045 or less; (ii) lower middle-income countries, with per capita GDP ranging from $1,046 to $4,095; (iii) upper middle-income countries, with per capita GDP ranging from $4,096 to $12,695; and (iv) high-income countries, with per capita GDP greater than $12,696. This strategy allows the use a linear model for all indicators, facilitating comparisons between groups and across countries.

We assume two scenarios to predict the evolution of each country in each health-related indicator yearly between 2021 and 2030: (i) the baseline scenario extrapolates the model for the whole decade ahead using the International Monetary Fund (IMF)’s pre-COVID-19 growth forecasts for each country. These were published in January 2020 to reflect the institution’s growth expectations in the last period of 2019. Thus, it did not consider any possible repercussion of COVID-19 pandemic [17]; (ii) the post-COVID-19 scenario adopts the IMF’s October 2021 growth forecasts update instead [18]. This specific report was chosen to represent the post-COVID period for it reflects a time where the epidemiological curve of the disease was already known, as well as the effective measures to contain and mitigate the multiple crises it prompted. Also, the forecast figures in this specific report did not anticipate the global economic repercussions of the Ukraine invasion by Russia in early 2022, freeing the estimate from this exogenous shock that would overlap with the pandemic. In other words, the two points were chosen so that most of the expectation changes were due to the direct and indirect impact of COVID-19.

In the estimation of both baseline and post-COVID scenarios, no changes in the countries’ levels of health expenditure and income inequality are assumed. In the case of health expenditures as a percentage of the GDP, assuming no changes throughout the decade do not compromise the global picture, since this is a political variable and thus exogenous by nature. Furthermore, in most cases, health expenditures increases during the pandemic were not sustained over the following years [19]. The hypothesis that the country Gini index will not change can be more heroic. This variable tends to evolve as result of many socioeconomic variables changes, including local policies and economic growth itself. Also, the evidence in the aftermath of the pandemic has shown significant increases in income inequality in some countries, even though the evidence in this matter is disputed [9, 20]. Some studies, however, suggest that the average level of income inequity has not changed significantly across countries over the last decades [21]. Hence, the best course of action, also considering the lack of accredited forecasts for the variable, was to assume it will remain the same over the decade.

Finally, the potential progress loss is defined as the difference between the forecasted values of each indicator generated by extrapolating these health-related curves for each country in post- and pre-pandemic scenarios.

## Results

Tables 2–4 present the estimation results for the health-related indicators in Eq 1. Overall, the models demonstrate a good fit for most of the 43 series, with one exception. Indicator 16.2.3, which measures the proportion of the population who experienced sexual violence, did not yield significant robustness measures for any parameter or the overall estimation. Notably, this lack of significance can be partly attributed to the limited data available, as only 46 countries reported data for this indicator.

**Table 2:** Global evolution curves: Health-related indicators.

Themes and variables	1. Maternal and reproductive health					2. Infant and neonatal health							
	ind311	ind312	ind371	ind372	ind223	ind221	ind222a	ind222b	ind321	ind322	ind3b1a	ind3b1b	ind3b1c
*ln* GDPpc	-0.4910***	0.2241***	0.2270***	-0.1746***	-0.1444***	-0.5842***	-0.3105***	0.2785***	-0.4491***	-0.4246***	0.0758***	0.1098***	0.0634
*ln* Gini	0.1885*	0.2180**	0.2016	0.0763	0.0719**	0.5606***	-0.2975	0.3641	-0.0947	0.0694	0.1257***	0.2702**	-0.3928**
*ln* Health exp. (% GDP)	-0.0818*	-0.0194	0.0836	-0.0607	-0.0499***	-0.0386	-0.3011***	0.1237	-0.0295	-0.0924***	-0.0042	0.1616***	0.1647**
Region dummy#*ln* GDPpc													
AMR	-0.1205***	0.0027	0.0254	-0.0274	-0.0505***	-0.0216	-0.1053***	0.0135	-0.0701***	-0.0367***	0.0016	0.0225**	-0.0018
CHN	-0.1524**	0.0188	0.1099**	-0.1665***	-0.0761***	-0.0907*	-0.0749	0.0392	-0.1411***	-0.1634***	0.017	0.0618*	-
EMR	-0.1313***	0.0009	0.0108	-0.1107***	-0.0129*	0.0139	0.0191	0.0451*	-0.0608***	-0.0328**	0.0038	0.0371***	-0.003
EUR	-0.2587***	0.0073	-0.0202	-0.1531***	-0.0378***	-0.0519***	-0.0568***	0.0369	-0.1458***	-0.1137***	0.0084*	0.0392***	-0.0131
IND	-0.0744	0.0156	0.0517	-0.1876***	0.03	0.0782	0.1247**	-0.0813	-0.0266	0.0149	0.0102	0.045	-0.1835***
SEAR	-0.0776***	-0.0124	0.0284	-0.0985***	0.008	0.0561**	0.0687***	-0.0412	-0.0767***	-0.0450**	0.0151**	0.0523***	-0.0927***
WPR	-0.1740***	0.0131	0.0195	-0.1180***	-0.0172***	-0.0217	-0.0173	0.0056	-0.0948***	-0.0793***	0.0088*	0.0309***	-0.012
Constant	9.5175***	1.4391***	0.8955	5.8722***	4.7815***	6.0956***	6.0676***	-2.2488**	8.5715***	6.8319***	3.2161***	1.7351***	4.4261***
N	569	563	308	582	566	367	363	360	760	760	747	555	361
FE (temporal)	yes	yes	yes	yes	yes	yes	yes	yes	yes	yes	yes	no	yes
r2_w	0.6684	0.3016	0.3091	0.2597	0.5045	0.5393	0.2626	0.0796	0.8312	0.7369	0.1335	0.0657	0.2354
r2_b	0.8916	0.4927	0.3428	0.739	0.7714	0.7296	0.6872	0.345	0.8519	0.8056	0.2869	0.3608	0.2538
r2_o	0.8949	0.4662	0.3589	0.7426	0.7757	0.6805	0.6567	0.3483	0.8738	0.8324	0.2835	0.2733	0.2497
Wald/F	2.10E+03	298.0273	137.5376	543.4762	929.2695	583.1513	329.9422	78.9588	3.80E+03	2.30E+03	154.1895	117.971	111.6817

Notes: (1) Due to temporal and transversal imbalances in the series, different estimation methods were applied. Whenever historical information was available, panel data estimation methods were adopted, allowing the identification of fixed effects of time and units. Five-year period averages were used to increase degrees of freedom in the estimate and to reduce the serial correlation identified in most series. For the other indicators, identified from the absence of correlation statistics (corr), we adopted cross-sectional estimates of ordinary least squares. (2) the sign—indicates that the dummy was excluded by collinearity; * p<0.1

** p<0.05

*** p<0.01

Source: Own elaboration

**Table 3:** Global evolution curves: Health-related indicators.

Themes and variables	3. Infectious diseases					4. Non-communicable diseases					5. Violence and injuries			
	ind331	ind332	ind333	ind334	ind335	ind211	ind341	ind342	ind352	ind3a1	ind1311	ind361	ind1611	ind1623
*ln* GDPpc	-1.6555***	-0.2701***	-1.3916***	-0.5782***	-2.6708***	-0.5888***	0.1024	-0.0125	0.6229***	-0.0624**	-0.4339*	-0.0294	-0.4311***	-0.3651
*ln* Gini	0.0577	-0.0451	2.4662***	-0.3789	3.2625**	0.169	-0.3218**	-0.1058	0.0531	0.1270*	0.3044	-0.0493	0.7216**	-0.0057
*ln* Health exp. (% GDP)	0.1662	0.0937	-1.1976***	-0.2888	-2.4397***	-0.0304	-0.0697	0.1903***	0.1	0.0688**	0.4578	-0.2543***	-0.3766**	-0.1717
Region dummy#*ln* GDPpc														
AMR	1.8238***	-0.1834***	-0.2611***	-0.1415***	0.2067**	-0.001	0.1255***	-0.0276	-0.0435	0.0136	0.0831	-0.0429***	0.1242***	0.0407
CHN	-	-0.0281	-1.1326***	-0.0108	0.6303**	-0.1171**	0.2073	-0.0201	-0.0098	0.1207***	0.1327	-0.0326	-	-
EMR	1.8094***	-0.1543***	-0.3753***	-0.1200***	-0.0517	0.0057	0.0963	-0.0573**	-0.2783***	0.0572***	-0.1364	-0.0308**	-0.0641	-0.1113
EUR	2.4371***	-0.1899***	-0.5914***	-0.0770**	-0.2715***	-0.0448***	-0.0472	0.0330*	-0.0407	0.0925***	-0.0819	-0.0912***	-0.0251	0.0162
IND	0.3658	0.0643	-0.1813	-0.1429	0.7624**	0.0212	0.4189**	0.0624	0.0397	0.1009**	0.5129	-0.0703	-0.0696	-0.0828
SEAR	0.9788**	0.0444	-0.3919***	-0.0897**	0.1927	-0.0054	0.1500**	-0.0529*	-0.2484***	0.1208***	0.1273	-0.0798***	-0.0613	-0.0502
WPR	2.2787***	-0.0423	-0.2209**	-0.0101	-0.0413	-0.0448**	-0.0104	0.0307	-0.0441	0.0899***	0.0983	-0.0914***	-0.0626	-0.0378
Constant	0.0858	7.6191***	8.8672***	7.2991***	28.1054***	7.0719***	7.6041***	2.3580***	-4.0208***	2.7866***	6.9512	4.2178***	3.5609**	4.1251
N	552	759	393	207	304	514	569	569	563	687	365	568	301	52
FE (temporal)	yes	yes	yes	no	no	yes	yes	yes	yes	yes	no	yes	yes	no
r2_w	0.3295	0.4479	0.3981	0.309	0.0331	0.3753	0.5276	0.1299	0.0866	0.7245	0.0247	0.1555	0.2758	0.1983
r2_b	0.4235	0.5698	0.6097	0.5293	0.7546	0.7488	0.1024	0.2148	0.5572	0.4852	0.2069	0.6041	0.6524	-
r2_o	0.414	0.57	0.6499	0.5549	0.7441	0.7291	0.1174	0.2018	0.5532	0.5366	0.1507	0.5621	0.6613	-
Wald/F	15.5012	702.0596	340.8342	190.6627	461.5193	593.9308	469.6846	101.2394	216.7214	1.50E+03	37.9891	289.0125	239.5728	0.8038

Notes: (1) Due to temporal and transversal imbalances in the series, different estimation methods were applied. Whenever historical information was available, panel data estimation methods were adopted, allowing the identification of fixed effects of time and units. Five-year period averages were used to increase degrees of freedom in the estimate and to reduce the serial correlation identified in most series. For the other indicators, identified from the absence of correlation statistics (corr), we adopted cross-sectional estimates of ordinary least squares. (2) the sign—indicates that the dummy was excluded by collinearity

* p<0.1

** p<0.05

*** p<0.01

Source: Own elaboration

**Table 4:** Global evolution curves: Health-related indicators.

Themes and variables	6. Environmental risks								7. Health systems and coverage							
	ind391	ind392	ind393	ind611	ind621a	ind621b	ind712	ind1162	ind1a2	ind381	ind382	ind3c1a	ind3c1b	ind3c1c	ind3c1d	ind3d1
*ln* GDPpc	-0.4000***	-0.8541***	-0.4085***	0.2702***	0.3708***	-0.5429***	0.4044***	-0.1296***	-0.2234***	0.2066***	-0.1895*	0.4798***	0.5774***	0.8653***	0.9323***	0.1134***
*ln* Gini	0.1794	0.7441	-0.1156	-0.0256	-0.0419	0.4132*	0.0692	-0.0192	-0.0282	0.0415	0.7674**	-0.2213	-0.4362***	-0.1183	0.4972	-0.0481
*ln* Health exp. (% GDP)	-0.3034***	-0.5986***	-0.0178	-0.1488***	-0.0798	-0.137	0.0556	-0.0948**	-	0.0614**	0.3962***	0.1936***	0.2414***	0.3481**	0.3717***	0.0796*
Region dummy#*ln* GDPpc																
AMR	-0.0183	-0.1801***	-0.1332***	0.0732***	0.0311	-0.0975***	0.1139***	-0.0555***	0.4396***	0.0287***	0.0519*	0.1541***	0.0086	0.1712***	0.0394	0.0228***
CHN	0.1133**	-0.2621***	0.0696	-	0.1022**	-0.2023*	0.0911	0.0442	0.3041**	0.0434***	0.2021**	0.1884***	0.0091	0.1709*	0.1605*	0.0529**
EMR	-0.0056	-0.1390***	-0.0634**	0.0694***	0.0332	-0.0696	0.1040***	0.0283*	0.3027**	0.0129**	0.0942***	0.1318***	-0.0209	0.1876***	0.1429***	0.0153*
EUR	0.0551***	-0.2537***	-0.0560**	0.0789***	0.0422**	-0.2702***	0.0914***	-0.0589***	0.2559***	0.0218***	0.0946***	0.1871***	0.0614***	0.1909***	0.0925***	0.0147*
IND	0.072	-0.0003	-0.1951**		0.0893	0.0802	0.1816**	0.0745	-0.2233	0.0199	0.1993*	0.1359*	0.036	0.2502**	0.3559***	0.0642**
SEAR	0.0247	-0.1079***	-0.1609***	0.0298	0.0513*	-0.1010**	0.1112***	-0.0242	0.2678**	0.0130**	0.0747*	0.1144***	0.0064	0.0996***	0.0611*	0.0254**
WPR	0.0132	-0.1693***	-0.0920***	0.0460***	0.0442**	-0.0601	0.0733***	-0.0957***	0.1745	0.0225***	0.034	0.1258***	0.0661***	0.1941***	0.1339***	0.0245***
Constant	7.5211***	8.4179***	4.7132***	1.2216**	0.1248	6.1385***	-1.0304**	4.8811***	1.8728***	1.4733***	-0.5773	-3.0133***	-1.1565	-9.376***	-11.485***	2.9794***
N	157	147	564	472	494	418	752	314	760	569	399	614	603	584	551	299
FE (temporal)	no	no	yes	yes	yes	yes	no	no	yes	yes	no	yes	yes	no	no	no
r2_w	0.5718	0.8721	0.2915	0.3206	0.43	0.586	0.2988	0.0262	0.1642	0.7066	0.0573	0.32	0.223	0.1772	0.198	0.2884
r2_b	-	-	0.5402	0.7906	0.6342	0.4363	0.7258	0.585	0.0643	0.8711	0.1267	0.8397	0.8062	0.8273	0.7665	0.4588
r2_o	-	-	0.5329	0.7913	0.6505	0.3914	0.7146	0.58	0.0846	0.8453	0.1068	0.8345	0.7841	0.7911	0.7369	0.4486
Wald/F	19.4957	92.6979	339.7715	457.559	460.7437	506.2221	635.4819	187.9434	8.4777	1.80E+03	33.8757	940.8548	755.2823	813.0939	566.0492	185.959

Notes: (1) Due to temporal and transversal imbalances in the series, different estimation methods were applied. Whenever historical information was available, panel data estimation methods were adopted, allowing the identification of fixed effects of time and units. Five-year period averages were used to increase degrees of freedom in the estimate and to reduce the serial correlation identified in most series. For the other indicators, identified from the absence of correlation statistics (corr), we adopted cross-sectional estimates of ordinary least squares. (2) the sign—indicates that the dummy was excluded by collinearity

* p<0.1

** p<0.05

*** p<0.01

Source: Own elaboration

Across all series, the association between per capita GDP and health-related indicators is statistically significant, either for all subsamples or at least for specific regional groups. In most cases, the signals and levels of robustness align with expected outcomes. The high R^2^ values (within, between, and overall) indicate that the models, particularly incorporating per capita GDP, health expenditures, and the Gini index, explain a substantial portion of the cross-country and time variability in these health indicators.

The magnitude of the association’s parameter (absolute value) reflects the expected progress in the indicator due to changes in per capita GDP. Conversely, when the parameter approaches zero, it suggests lesser conditional expectation of indicator changes linked to improvements in socioeconomic development, as represented by per capita GDP. In such cases, other local factors and uncorrelated omitted variables might exert greater influence. For instance, an indicator 3.1.1 parameter of -0.49 indicates that a 1% increase in per capita GDP corresponds to a reduction of approximately 0.5% in maternal mortality worldwide.

To account for regional differences in elasticity, we introduced multiplicative regional dummies for the six WHO regions—African (AFR), Americas (AMR), Southeast Asian (SEAR), European (EUR), Eastern Mediterranean (EMR), and Western Pacific (WPR)—along with China (CHN) and India (IND) due to their unique population size and characteristics. Except for cases indicated in Tables 2–4, the baseline is the African region. To exemplify, taking again indicator 3.3.1, 1% increases in the GDP are expected to be associated with an additional 0.26% and 0.17% reductions (that is, -.75 and -.66), respectively, in the European and Eastern Mediterranean regions (one should note that WHO regions differ significantly from that of usual geographical regions–please refer to S1–S4 Tables for references).

Out of the 43 indicators, four showed no significant regional differences: births assisted by qualified personnel (3.1.2), overweight (2.2.2b), death rate from natural disasters (13.1.1), and the proportion of the population who experienced sexual violence (16.2.3). However, in the remaining 39 indicators, regional variability in the *GDP*_pc_ parameter was evident to varying degrees. Notably, certain indicators, such as HIV/AIDS incidence (3.3.1) and homicides (16.1.1), exhibited outlier regions with notable differences. Additionally, for some series, regional characteristics were so dominant that distinct regional evolution curves were evident instead of a global trend. Examples include premature deaths from noncommunicable diseases (3.4.1), suicide rate (3.4.2), and deaths due to road traffic injuries (3.6.1).

The results for three indicators is of particular concern. Surprisingly, the elasticity of children with overweight (2.2.2b) and alcohol consumption (3.5.2) in relation to per capita GDP is positive and significant. This indicates that increases in development levels may worsen these health conditions, contrary to expectations. Another indicator, premature deaths from noncommunicable diseases (NCD) (3.4.1), also falls into this group, as coefficients for India, SEAR, and the Americas regions are all positive and significant. Although the global variance of this indicator rendered the overall parameter insignificant, the positive regional coefficients are noteworthy.

The Gini index showed significance in 15 indicator series. With the exception of births assisted by qualified health personnel (3.1.2), where increases in inequality are associated with increased coverage, the estimates align with expectations. Income inequality played a crucial role in exacerbating the percentage of children with stunted growth (2.2.1), children’s immunization coverage (indicators 3.b.1a-b), Malaria incidence (3.3.3), people affected by neglected tropical diseases (3.3.5), mortality due to NCD (3.4.1), prevalence of smoking (3.a.1), homicides (16.1.1), and impoverishing out-of-pocket health expenditures (3.8.2). In many cases, income inequality emerged as the primary factor influencing these phenomena.

Health expenditure significantly impacted 23 out of 43 indicator series. In most cases, the results align with expectations, except for drinking water managed services (3.6.1), where the variable may be reflecting the effect of omitted factors. An unexpected positive association between health expenditure and suicide rates (3.4.2) and the prevalence of smoking (3.a.1) was found, but these results can still be understood in the context of the variables’ dynamics. Notably, health expenditure played a significant role in explaining the global dynamics of health systems and coverage indicators. Other noteworthy associations include children wasting (2.2.2a), immunization (indicators 3.b.1b-c), death rates from natural disasters (13.1.1), mortality attributed to pollution (3.9.1), and exposure to unsafe water, sanitation, and hygiene services (3.9.2).

S5 Table shows the *GDP*_pc_ elasticities for the estimation of Eq 1 for subsamples of countries at the four income groups of World Bank (the results for covariates and regional parameters, as well as the robustness measures are available upon request). Overall, the robustness measure confirm the good results of these subsample estimates, even in the case of indicators 2.2.2b, 3.b.1b, 13.1.1 and 3.8.2, for which the global *GDP*_pc_ elasticity was not significant for any income groups. Regional parameters and the other covariates seem to explain the indicator’s variance significantly in these cases. Some estimations are omitted due to missing data for the countries in the income groups. Being conservative in the measurement of the loss, we also opted to dismiss all indicators for which less than 40% of the countries in the income group did not report the indicator (these are indicated in S5 Table with a trace).

For indicators of children stunting (2.2.1), pregnant women with anemia (2.2.3), children and neonatal mortality (3.2.1, 3.2.2), mortality due to NCD (3.4.1) and practice of open defecation (6.2.1b) the difference between groups are not statistically significant, suggesting that for these indicators, the general levels of development do not influence in the association between increases in *GDP*_pc_ and improvements in health conditions.

For the remaining indicators, however, there are important differences in the estimated coefficient across the income groups, even though these are all within a reasonable range. The only two extreme cases, for which the coefficient signals are inverted in different groups are: road traffic deaths (3.6.1) and concentration of fine particulate matter (11.6.2), where the *GDP*_pc_ elasticity of the indicator is positive for lower income countries and negative for high-income countries. In both cases, these are within expectations.

Preston’s hypothesis are more evident in some indicators associated with assess and coverage of health services and basic infrastructure, such as the population that uses safely managed drinking water and sanitation services (6.1.1, 6.2.1a), prevalence of undernourishment (2.1.1), coverage of essential health services (3.8.1), births assisted by qualified personnel (3.1.2), DPT3 immunization (3.b.1a), and the number of health professional per 100,000 inhabitants (3.c.1a-d), where one may verify diminishing returns to changes in *GDP*_pc_ as we skip to higher income groups.

For some health themes, the impact of increments in per capita income on the indicator is heavily concentrated in low-income countries, as in the case of family planning (3.7.1), children wasting (2.2.2a), vaccine coverage (3.b.1a-b), HIV infections (3.3.1) and alcohol consumption (3.5.2). That is, the effect of increments in the country´s per capita income on the indicators’ improvement are higher the lower the income. For the first two, however, this result can be associated with the excessive number of missing information for high-income countries.

In other health topics, the effect of improvement in income on the indicator is concentrated in higher income groups, such as mortality rates attributable to pollution and unintentional poisoning (3.9.1 and 3.9.3), health expenditures (1.a.2) and homicides (16.1.1). In all cases, the effect is particularly stronger in high-income countries, an indicative that in these topics, progress due to improvements in general levels of income are not common until the country reaches the higher levels of development.

There are also indicators such as communicable diseases where the effect of increments in per capita income on the indicator peaks for middle-income countries (inverted U pattern) and those for which the peaks are in the extreme income groups (U pattern). In the first category are mortality due to unsafe water, sanitation and hygiene (WASH) services (3.9.2), capacity of the IHR and health emergency preparedness (3.d.1), and Malaria incidence (3.3.3), even though, for the latter, this can be the result of missing information for high-income countries. The U pattern is found for indicators of Tuberculosis incidence (3.3.2) and suicide rates (3.4.2).

These differences in the response to variations in per capita GDP influence the evolution of these health-related indicators in countries at different income groups. Even if the scenarios of economic growth are resembled, the projected figures for countries at lower/higher income levels are different. This is illustrated in S1–S4 Tables, which show the predicted accumulated losses in the evolution of each of the 7 health themes by 2030 in each country of the sample in response to the economic growth revisions since the pandemic outbreak. The tables also present the each of the scenarios considered for the projection, including the values assumed for the control variables.

To illustrate the potential inequalities in impact worldwide, Table 5 summarize the accumulated losses by 2030 in the seven health themes for the four income groups. The expected losses in GDP are disproportionately greater in poorer countries, with a mean of 42% and 28% in low- and lower middle-income countries, respectively, compared to 15% and 7% in high- and upper middle-income countries, respectively.

**Table 5:** Mean estimated losses from the economic crisis generated by COVID-19 by 2030: Country income groups.

Indicators		Country Groups^1^			
		Low income	Lower-middle income	Upper-middle income	High income
Annual average growth rate 2010s		4.32	4.16	3.16	2.31
GDPpc (2019)		2,060.02	6,952.56	16,489.29	46,449.74
Growth rate Pre-COVID^2^		5.20	4.27	3.07	2.12
Forecasted per capita GDP pre-Covid (2030)^3^		3,800.46	11,519.97	23,844.50	58,733.41
Growth rate Post-COVID^2^		4.36	3.45	2.71	2.02
Forecasted per capita GDP post-Covid (2030)^3^		2,931.23	9,579.79	21,315.85	55,512.59
GDPpc loss as a proportion of the initial GDPpc		-42%	-28%	-15%	-7%
Average GINI index^4^		41.3	38.2	41.0	35.6
Average Health expenditure (% GDP)^5^		5.85	5.75	7.14	9.00
**Health-related indicators**	1. Maternal and reproductive health	-16.78%	-8.90%	-2.46%	-3.31%
	2. Newborn and child health	-13.36%	-9.26%	-5.23%	-2.40%
	3. Infectious diseases	-33.77%	-30.69%	-29.50%	-6.37%
	4. Noncomunicable diseases	-6.30%	-1.44%	-1.99%	-0.42%
	5. Injuries and violence	-18.23%	3.53%	-5.92%	-1.97%
	6. Environmental risks	-12.72%	-14.95%	-5.63%	-4.61%
	7. Health systems and coverage	-14.23%	-8.97%	-3.05%	-1.38%
**Average change (health-related indicators)**		**-16.48%**	**-10.10%**	**-7.71%**	**-2.9%**

Notes

^1^ World Bank classification.

^2^ IMF growth projections.

^3^ 2015 USD$ constant.

^4^ World Bank.

^5^ WHO estimates.

Source: own elaboration

As expected, the average losses decrease with the income level, with low-income countries experiencing losses more than 5.5 times higher than high-income countries. This pattern is consistent across most themes, with low-income countries leading the losses in their respective indicators. However, in relative terms, the themes of injuries and violence and maternal and reproductive health stand out, showing much higher losses than expected from the economic downturn due to the high GDP elasticity of the indicators in these income groups. Infectious diseases represent the worst case, with expected losses surpassing 33%.

Middle-income countries are also significantly affected, particularly in the theme of infectious diseases, where losses reach around 30%. For lower-middle income countries, environmental risks, maternal, child, and newborn health, as well as health systems and coverage, are areas of concern, with losses averaging around 10%. In comparison, noncommunicable diseases and injuries and violence can be a more pressing issue for upper-middle income countries compared to countries with lower income levels. High-income countries see the most significant losses in infectious diseases, environmental risks, and maternal and reproductive health. However, except for the latter, the losses in these themes are considerably lower compared to other income groups.

**Fig 1:**
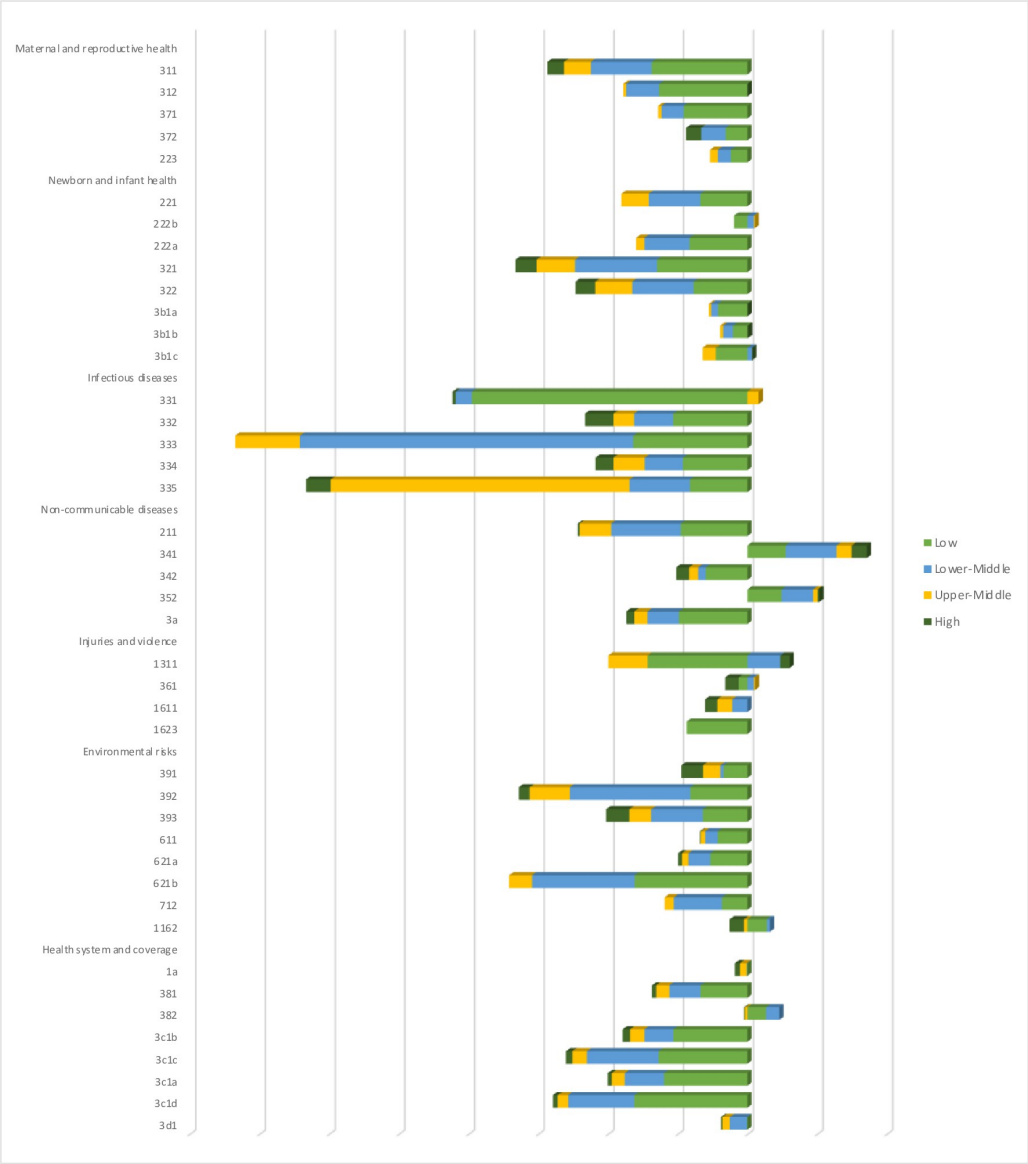
Mean cumulative losses by 2030 of the health-related indicators: Income groups. Notes: The horizontal axis is intentionally suppressed not generate misinterpretations of the stacked bars. Source: own estimations.

Fig 1 presents the average cumulative losses by 2030 for each health-related indicator, detailing the results from the previous table and identifying which indicators are responsible for the losses in each health theme and income groups. In low-income countries, HIV infections (3.3.1), Malaria (3.3.3), and NTD (3.3.5) are major contributors to the concerning outcomes in infectious diseases.

**Fig 2:**
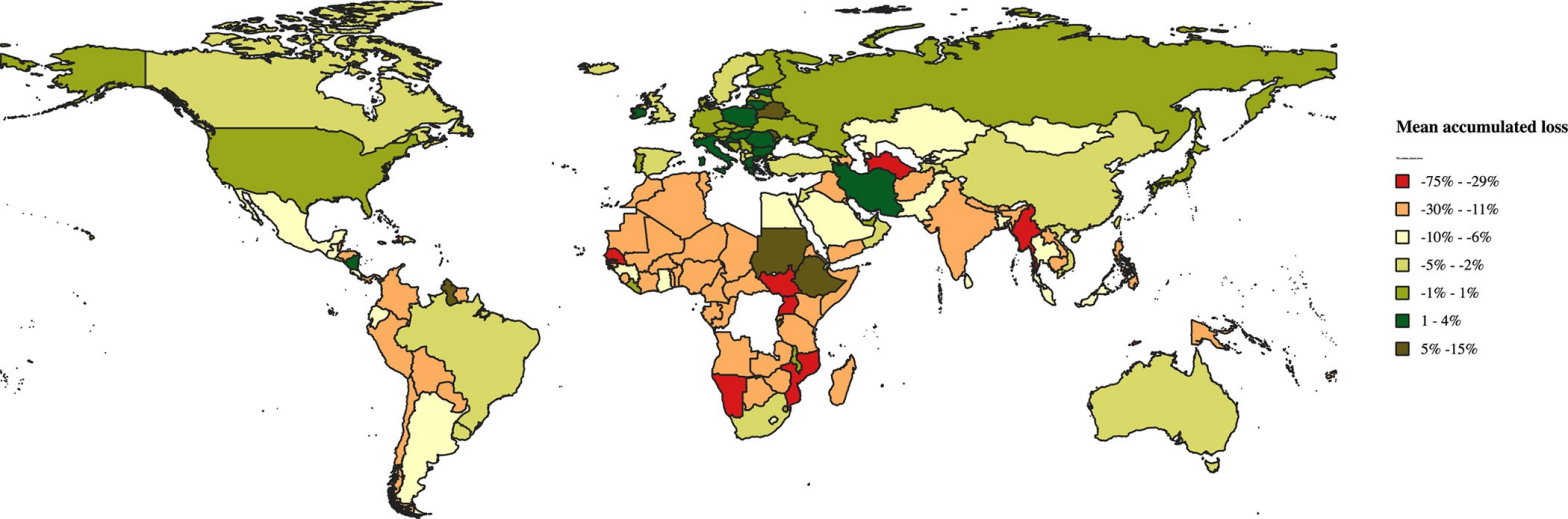
Mean cumulative losses by 2030 in all health-related indicators: Countries. Note: National losses calculated from the mean loss for health-related indicators in the country. Source: Own elaboration. Original shapefile retrieved from the World Bank, available under a CC BY 4.0 license. For illustrative purposes only. For more information, see https://datacatalog.worldbank.org/search/dataset/0038272/World-Bank-Official-Boundaries.

The disparities shown in Fig 2 are even more pronounced than those in the previous table, with low-income countries dominating the losses across most indicators. Notably, NCDs (3.4.1) and alcohol consumption (3.5.2) are expected to improve in all income groups due to the inverted sign of improvement found for these indicators. Additionally, certain topics, such as deaths from disasters (13.1.1), fine particulate matters (11.6.2), and impoverishing out-of-pocket expenditures in health (3.8.2), are expected to see improvements in specific income groups.

The global disparities in expected losses are even more evident when considering the countries’ perspective (see the last columns of S1–S4 Tables). While the average loss of potential in the set of health indicators is approximately 8.4%, some countries, like Turkmenistan and Myanmar, are projected to face up to 9 times worse results, while others have had their prospects revised upward, including several high-income countries. Fig 2 illustrates these results, presenting the mean accumulated effect by 2030 on the set of health indicators for countries in the sample. The most significant losses are concentrated in Africa, the Middle East, Southern Asia, and Latin America. Positive results due to upward revisions are evident in Northern Africa (Libya and Sudan), Iran, Eastern Europe, among others.

## Discussion

The discrepancies in COVID-19 mortality between countries can be attributed to a multitude of factors. Demographic elements, such as population density, the proportion of elderly individuals (age 80 or older), and the urban population ratio, have been identified as influential [22]. Additionally, economic, social, and political efforts, encompassing mitigation measures, mobility restrictions, and healthcare interventions, play a significant role in shaping the COVID-19 burden [23–25]. Social, economic, and ethnic disparities have also emerged in COVID-19 burdens across different countries [26–28].

The economic impact of the pandemic has been highly uneven across global economies. While virtually all countries experienced disruptions in production chains, leading to a 3.5% global GDP drop in 2020 –the most severe economic recession since the 1930s –the extent of economic contraction varied significantly. Latin America and the Caribbean saw a 7% downturn in GDP, similar to Europe’s 6.3% decline, whereas developing countries in Asia experienced only a 0.8% decline. Swift health measures, social protection programs, and other mitigation policies helped mitigate the pandemic’s adverse effects in some countries [29].

These disparities are expected to be exacerbated by unequal access to vaccines and varying fiscal capacities among wealthier nations, many of which have already committed to major public investments for economic recovery. Some countries, like the USA, have witnessed improved economic prospects for the next decade. In contrast, heavily indebted poorer economies may experience heightened inequalities globally, particularly impacting the health-related targets of the 2030 Agenda, as demonstrated in this study [30]. According to the IMF projections used in this research, low-income countries are projected to face losses in economic output that are, on average, 2.8 times greater than upper middle-income countries and six times greater than high-income countries, despite some high-income countries being significantly impacted by the crisis in 2020–2021.

The anticipated loss in health indicators due to changes in economic prospects is projected to be most significant in low-income countries, particularly in the areas of infectious diseases, violence and injuries, and maternal and reproductive health. Lower middle-income countries are expected to experience greater losses in indicators of infectious diseases and environmental risks, where economic gains tend to influence indicator improvements more strongly. Historically, these health issues have posed significant challenges for developing countries, where healthcare systems and socioeconomic inequalities can negatively impact health resource distribution [31]. The weaker macrosocial determinants and socioeconomic contexts in low-income countries may exacerbate the pandemic’s impact on population health [32]. Increased unemployment and job insecurity resulting from the economic crisis can also heighten demand for public services, particularly in the health sector, further emphasizing the importance of expanding access and improving health infrastructure to minimize the short and long-term impact of economic and epidemiological crises.

Universal health coverage emerges as a central issue for global health [33]. The study indicates that significant disparities exist between low- and high-income countries in the areas of health systems and coverage. Recent global agendas have emphasized the call for universal health coverage, supported in part by the explicit inclusion of universal coverage in target 3.8 of the SDGs. Although there has been a substantial improvement in the indicator of effective universal health coverage from 1990 to 2019, disparities still exist between nations, indicating potential exacerbation by 2030 [34].

This paper underscored the potential losses due changes in the near future economic perspectives, but the COVID-19 pandemic impacts both the exposure factors, that is, the health determinants, and the health effects: situations of morbidity and mortality. The latter effects are still being tabulated at the global level, and thus far there is no clear scenario for the impact´s size, but the effects will definitely lead to significant worsening in all the target indicators, whether directly from the effect of reducing coverage in health services that were already at their limit (saturated) during the pandemic, limiting their action on other fronts [35], and indirectly, as in the case of mortality from violence, traffic accidents, and other risk factors such as alcohol abuse, smoking, and psychological disorders caused by the combination of the economic recession and social isolation. Preliminary studies indicate that alterations in lifestyle, with the increase in physical inactivity and the high incidence of mental disorders due to social isolation and the economic crisis, will be further reflected in noncommunicable diseases in the medium and long term [36, 37]. Interestingly, noncommunicable diseases are those with the least expected impact in this study, due to their lower per capita GDP elasticity.

This study presents other noteworthy limitations. Firstly, the presence of missing data for some indicators in specific income groups reduces the accuracy of the global evolution curves and hampers cross-country comparisons, particularly among those at different income levels. The discrepancies in the number of indicators across income groups and even among countries within the same group complicates the resolution of this issue. Excluding some indicators to prioritize those with complete data coverage could potentially lead to even greater distortions, obscuring crucial health phenomena. Keeping the estimations for all cases in which at least 40% of the sample was available increases the significance of within group comparisons.

Additionally, the global evolution curves may underplay the influence of local-specific factors, including epidemiological characteristics and the coverage and quality of the local health system and services. To mitigate this concern, we incorporated local time-invariant effects and regional dynamic effects into the model. Nevertheless, the ability of global curves to represent country-specific phenomena depends on the significance of transnational factors in the determination of the health topic under study. For instance, if the set of health interventions and outcomes for a specific disease is consistent across the world, a considerable share of cross-country differences in mortality indicators could be attributed to variations in intervention coverage and treatment availability (technology) across countries. As a result, this study’s approach should be viewed as complementary to the analysis of individual countries’ trajectories.

An additional concern associates with the measure of the pandemic impact adopted. The differences between the IMF’s growth projections in January 2020 and October 2021 obviously do not discriminate between the COVID-19 and other national and international shocks. In any case, the pervasiveness of its consequences in society makes of the pandemic the lead exogenous change in the period, so that the differences in the figures between these specific reports can be seen as a good proxy for its impact. It is also worth mentioning that the long-standing methodology adopted by the IMF captures the main economic forces (and determinants) in play at the time, thus reflecting not only the effectiveness of the sanitary and mitigation policies adopted in response to the pandemic, which varied considerably around the world (see [9] for a compilation), but also their repercussions in other societal areas in each country.

## Conclusions

The COVID-19 pandemic has delivered a devastating blow to global health, with its economic repercussions throughout the 2020 decade threatening to widen existing disparities between wealthy and low-income countries. This study investigated the potential impact of these economic disruptions on progress towards the health-related SDG indicators.

The analysis was based on transnational trends captured by a regression model that extends the Preston’s curve hypotheses to include other key health determinants. The approach proved successful, showing significant parameters for the association between the GDP_pc_ and most health indicators. The global curves also revealed important characteristics of the evolution of each health-related indicator.

Comparisons between a baseline projection and a post-COVID-19 scenario revealed a concerning trend: low-income countries are expected to suffer disproportionally from the economic consequences of the pandemic. While low-income countries may face an average economic growth loss of 42% compared with the baseline scenario, the average loss in in high-income countries is of 7%. These disproportional figures are shown to exacerbate global health inequalities revealed by the curves. The expected progress loss in infectious diseases in low-income countries, for instance, is an average of 33.8%, against a mean of 6.4% in high-income countries. Infectious diseases are followed by injuries and violence, maternal and reproductive health, health systems coverage and maternal and infant health indicators as those that will face the stronger blow. Low-income countries can expect an average progress loss of 16.5% across all health indicators assessed, whereas in high-income countries the estimated loss is of 3%. The disparity across countries is even more pronounced, with cases where the estimated progress loss is as high as nine times worse than the average loss of 8.4%. Conversely, countries with greater fiscal capacity are likely to fare much better under the circumstances, despite their worse death count, in many cases.

It is essential to underscore that while we observed a consistent association between increases in per capita Gross Domestic Production and improvements in the health indicators, this empirical relationship does not imply a causal link. Indeed, multiple factors contribute to the explanation of the health phenomena in each country, and economic growth can be just a second order or indirect factor. This means that, against what some authors defended in the past [38], economic growth cannot be effectively used to fight health issues directly.

A more in-depth investigation into the economic and social determinants of health is critical for facilitating effective action [2]. Future research should thus explore additional local-level factors that may influence health outcomes alongside global economic trends. A more detailed account of sanitary and other mitigation policies measures implemented by each country can also be contrasted with the epidemiological profile and evolution of the disease in each country to provide more insights on why some of them managed to perform so much better than others in containing the spread of the disease and their socioeconomic consequences.

The study’s findings have significant implications for international public health policy. To mitigate worldwide disparities in health, international cooperation and targeted support are crucial to strengthen healthcare systems in low-income countries and ensure equitable progress towards a healthier future for all. We contend that the SDGs serve as a vital tool for tackling global development challenges. It offers internationally negotiated targets, indicators already consolidated within a monitoring model, and a comprehensive and inclusive agenda for fighting inequalities. By embracing the principles of the 2030 Agenda, we can pave the way for significant progress in addressing global health disparities and promoting sustainable development.

## Supporting information

S1 Table Economic scenarios and mean estimated losses by health themes in 2030: Low-income countries. Notes: * Gini estimates. GDPpc and growth rates from IMF. Source: own elaboration.
(PDF)

S2 Table Economic scenarios and mean estimated losses by health themes in 2030: Lower middle-income countries. Notes: * Gini estimates. GDPpc and growth rates from IMF. Source: own elaboration.
(PDF)

S3 Table Economic scenarios and mean estimated losses by health themes in 2030: Upper middle-income countries. Notes: * Gini estimates. GDPpc and growth rates from IMF. Source: own elaboration.
(PDF)

S4 Table Economic scenarios and mean estimated losses by health themes in 2030: High-income countries. Notes: * Gini estimates. GDPpc and growth rates from IMF. Source: own elaboration.
(PDF)

S5 Table Per capita GDP elasticity of the SDG indicator: By income strata. Notes: Panel data estimations of Eq (1) by subsamples of the income strata. Significance level * p<0.1; ** p<0.05; *** p<0.01. Source: own estimations.
(PDF)

S1 Dataset (ZIP)
