# Supplementary material for: Post-COVID-19 health inequalities: Estimates of the potential loss in the evolution of the health-related SDGs indicators
Source: PLoS One. 2024 Jul 24;19(7):e0305955. doi: 10.1371/journal.pone.0305955 (PMC11268624; doi:10.1371/journal.pone.0305955)
Supplement: S1 Table — Notes: * Gini estimates. GDPpc and growth rates from IMF. Source: own elaboration. (PDF) [file pone.0305955.s001.pdf]

S1 Table – Economic scenarios and mean estimated losses by health themes in 2030: low-income countries

		**Economic scenarios**						**Fixed covariates**		**Accumulated losses in the decade**							
**Country**	**WHO Region**	Annual average growth rate (2010-2019)	GDP per capita (2019)	Growth rate Pre-COVID (2020-2030)	GDP per capita pre-Covid (2030)	Growth rate Post-COVID (2020-2030)	GDP per capita post-Covid (2030)	GINI index	Average Health expenditure (% GDP)	1. Maternal and reproductive health	2. Newborn and child health	3. Infectious diseases	4. Non-comunicable diseases	5. Injuries and violence	6. Environ-mental risks	7. Health systems and coverage	**Mean general loss**
**Afghanistan***	EMR	4.82	$ 2,439.68	5.04	$ 4,232.81	-2.35	$ 3,531.30	59	15.53	-12.5%	-8.5%	-24.9%	-7.4%	-13.1%	-10.2%	-12.0%	**-12.7%**
**Benin**	AFR	4.78	$ 3,283.84	6.74	$ 6,899.16	6.15	$ 5,214.51	42.8	2.59	-19.2%	-15.6%	-37.0%	-9.2%	-24.2%	-13.3%	-17.0%	**-19.4%**
**Burkina Faso**	AFR	6.02	$ 2,178.32	6.00	$ 4,287.72	5.14	$ 3,092.95	47.3	6.72	-22.2%	-17.3%	-44.9%	-11.1%	-25.7%	-15.8%	-19.3%	**-22.3%**
**Burundi**	AFR	2.22	$ 751.76	0.50	$ 806.55	4.05	$ 944.16	38.6	6.5	11.6%	7.6%	15.2%	0.7%	10.8%	6.3%	13.0%	**9.3%**
**Central African Rep.**	AFR	-0.20	$ 945.18	4.99	$ 1,665.10	3.95	$ 1,257.41	56.2	9.4	-19.2%	-15.7%	-24.7%	-4.9%	-2.8%	-15.4%	-20.5%	**-14.7%**
**Chad**	AFR	3.43	$ 1,587.07	4.17	$ 2,546.81	3.10	$ 1,813.35	37.5	5.41	-23.1%	-19.7%	-47.2%	-11.7%	-20.3%	-18.8%	-17.3%	**-22.6%**
**Comoros**	AFR	3.20	$ 2,978.79	3.55	$ 4,469.85	3.51	$ 3,430.51	45.3	5.35	-18.1%	-15.0%	-34.6%	-3.2%	-22.7%	-12.5%	-16.3%	**-17.5%**
**Congo (DRC)**	AFR	6.23	$ 1,097.95	4.39	$ 1,794.45	5.64	$ 1,623.40	42.1	4.05	-7.0%	-5.4%	-11.5%	-2.8%	-5.4%	-5.4%	-6.9%	**-6.3%**
**Eritrea***	AFR	5.26	$ 1,761.13	4.84	$ 2,976.82	3.46	$ 2,271.84	37.6	4.09	-21.3%	-14.1%	-35.5%	-3.3%	-2.7%	-12.8%	-19.8%	**-15.6%**
**Ethiopia**	AFR	9.63	$ 2,641.01	6.69	$ 5,388.35	4.02	$ 5,849.90	35	3.48	6.0%	4.0%	8.3%	2.0%	5.9%	4.4%	6.4%	**5.3%**
**Gambia**	AFR	2.88	$ 2,225.31	5.10	$ 3,973.99	5.26	$ 3,174.52	38.8	2.61	-15.5%	-11.6%	-28.4%	-7.0%	-18.9%	-9.9%	-14.2%	**-15.1%**
**Guinea**	AFR	6.03	$ 2,405.89	5.17	$ 4,302.58	5.61	$ 3,688.96	29.6	4.04	-10.7%	-8.5%	-18.4%	2.5%	-1.5%	-6.9%	-10.2%	**-7.7%**
**Guinea-Bissau**	AFR	4.03	$ 2,330.24	5.21	$ 4,167.26	4.17	$ 3,143.63	34.8	8.41	-19.3%	-15.7%	-37.3%	-3.5%	-21.6%	-15.5%	-17.1%	**-18.6%**
**Haiti**	AMR	1.29	$ 2,989.79	1.47	$ 3,518.54	0.75	$ 2,950.10	41.1	3.22	-11.8%	-8.5%	-21.7%	-4.1%	-9.4%	-7.6%	-11.1%	**-10.6%**
**Liberia**	AFR	3.16	$ 1,553.07	2.48	$ 2,154.26	4.50	$ 2,116.53	35.3	9.5	-1.3%	-0.9%	-1.9%	-0.5%	-1.3%	-0.8%	-1.3%	**-1.1%**
**Madagascar**	AFR	2.95	$ 1,585.15	4.89	$ 2,837.69	3.74	$ 2,040.44	42.5	3.88	-22.5%	-17.5%	-45.4%	-11.3%	-26.0%	-18.2%	-19.4%	**-22.9%**
**Malawi**	AFR	4.36	$ 1,411.37	6.24	$ 1,927.02	4.69	$ 1,912.93	41.6	5.43	-0.5%	-0.4%	-0.8%	-0.2%	-0.5%	-0.4%	-0.5%	**-0.5%**
**Mali**	AFR	4.40	$ 2,392.47	4.84	$ 4,156.05	4.34	$ 3,097.03	36.1	4.31	-20.1%	-15.5%	-39.3%	-9.8%	-25.7%	-15.4%	-17.7%	**-20.5%**
**Mozambique**	AFR	5.56	$ 1,281.78	9.42	$ 3,421.12	6.83	$ 2,156.81	54	7.62	-31.1%	-25.1%	-71.1%	-17.5%	-44.2%	-23.5%	-25.0%	**-33.9%**
**Nepal**	SEAR	5.01	$ 3,972.63	5.19	$ 7,049.25	4.23	$ 5,701.50	32.8	5.17	-14.5%	-10.9%	-31.0%	-6.0%	-17.1%	-11.9%	-14.3%	**-15.1%**
**Niger**	AFR	6.17	$ 1,224.34	7.00	$ 2,682.93	7.37	$ 2,061.21	37.3	6.2	-18.1%	-13.8%	-34.4%	-3.2%	-20.0%	-13.7%	-16.2%	**-17.0%**
**Rwanda**	AFR	7.17	$ 2,268.35	7.66	$ 5,157.27	5.91	$ 3,635.54	43.7	7.32	-23.8%	-18.6%	-49.0%	-12.1%	-31.5%	-18.2%	-20.3%	**-24.8%**
**Senegal**	AFR	4.81	$ 3,361.46	8.14	$ 8,293.94	5.54	$ 5,020.64	38.1	5.15	-33.8%	-27.6%	-80.2%	-19.6%	-49.3%	-27.6%	-26.6%	**-37.8%**
**Sierra Leone**	AFR	5.07	$ 1,709.30	4.69	$ 2,875.84	3.94	$ 2,255.74	35.7	8.76	-16.7%	-12.6%	-31.2%	-7.7%	-20.6%	-13.3%	-15.1%	**-16.8%**
**Somalia***	AFR	2.36	$ 915.54	3.47	$ 1,371.44	3.41	$ 1,082.06	39.7	8.5	-16.3%	-15.7%	-30.3%	-9.0%	-2.3%	-12.2%	-9.1%	**-13.6%**
**South Sudan**	AFR	-5.08	$ 827.11	5.21	$ 1,594.72	3.84	$ 1,018.15	44.1	5.25	-34.7%	-26.9%	-68.4%	-1.6%	-38.0%	-22.8%	-24.5%	**-31.0%**
**Togo**	AFR	5.72	$ 2,121.25	5.40	$ 3,863.32	5.80	$ 3,321.62	42.7	5.96	-10.5%	-7.7%	-18.1%	-4.4%	-12.2%	-8.2%	-10.0%	**-10.2%**
**Uganda**	AFR	5.55	$ 2,564.78	8.80	$ 6,565.84	5.90	$ 3,888.01	42.7	3.96	-35.2%	-30.9%	-85.4%	-9.0%	-52.1%	-28.6%	-27.4%	**-38.4%**
**Tanzania (URT)**	AFR	6.67	$ 2,725.39	6.34	$ 5,431.00	5.57	$ 4,025.89	40.5	3.75	-20.4%	-15.7%	-40.2%	-10.0%	-26.2%	-15.7%	-18.0%	**-20.9%**
**Zimbabwe**	AFR	6.08	$ 2,270.53	2.28	$ 3,602.04	2.59	$ 2,616.20	47.3	3.43	-21.8%	-16.9%	-43.7%	-4.2%	-28.3%	-17.7%	-19.0%	**-21.6%**

Notes: * Gini estimates. GDPpc and growth rates from IMF.

Source: own elaboration
