# Supplementary material for: Post-COVID-19 health inequalities: Estimates of the potential loss in the evolution of the health-related SDGs indicators
Source: PLoS One. 2024 Jul 24;19(7):e0305955. doi: 10.1371/journal.pone.0305955 (PMC11268624; doi:10.1371/journal.pone.0305955)
Supplement: S2 Table — Notes: * Gini estimates. GDPpc and growth rates from IMF. Source: own elaboration. (PDF) [file pone.0305955.s002.pdf]

S2 Table – Economic scenarios and mean estimated losses by health themes in 2030: lower middle-income countries

		**Economic scenarios**						**Fixed covariates**		**Accumulated losses in the decade**							
**Country**	**WHO Region**	Annual average growth rate (2010-2019)	GDP per capita (2019)	Growth rate Pre-COVID (2020-2030)	GDP per capita pre-Covid (2030)	Growth rate Post-COVID (2020-2030)	GDP per capita post-Covid (2030)	GINI index	Average Health expenditure (% GDP)	1. Maternal and reproductive health	2. Newborn and child health	3. Infectious diseases	4. Non-comunicable diseases	5. Injuries and violence	6. Environ-mental risks	7. Health systems and coverage	**Mean general loss**
**Angola**	AFR	2.23	$ 7,043.65	3.45	$ 10,575.96	2.34	$ 7,353.74	51.3	2.91	-14.2%	-15.7%	-51.7%	1.0%	12.5%	-27.5%	-14.2%	**-15.7%**
**Armenia**	EUR	4.49	$ 13,637.87	4.53	$ 21,813.05	3.29	$ 19,332.15	26.5	12.24	-5.7%	-5.0%	-7.9%	-0.7%	-0.3%	-7.0%	-6.1%	**-4.7%**
**Bangladesh**	SEAR	6.76	$ 5,113.78	7.31	$ 11,144.98	6.54	$ 9,523.60	32.4	2.63	-6.8%	-6.2%	-23.9%	-1.2%	1.1%	-8.9%	-6.9%	**-7.5%**
**Bhutan**	SEAR	6.15	$ 11,639.56	6.55	$ 23,773.05	4.48	$ 17,535.51	37.4	4.37	-13.3%	-12.7%	-48.4%	9.1%	1.6%	-17.9%	-12.6%	**-13.5%**
**Bolivia**	AMR	4.65	$ 8,757.26	3.71	$ 13,390.74	2.56	$ 10,416.83	42.2	7.86	-11.1%	-11.4%	-38.0%	-1.7%	2.9%	-19.5%	-11.4%	**-12.9%**
**Cabo Verde**	AFR	2.75	$ 7,167.75	4.98	$ 12,294.55	4.10	$ 10,054.66	42.4	6.02	-8.1%	-10.1%	-11.0%	-1.3%	1.1%	-14.1%	-10.2%	**-7.7%**
**Cambodia**	WPR	7.03	$ 4,638.22	6.57	$ 9,396.28	5.14	$ 7,515.96	45.4	7.51	-10.1%	-9.7%	-31.6%	-1.3%	8.2%	-16.2%	-10.5%	**-10.2%**
**Cameroon**	AFR	4.50	$ 3,642.28	5.15	$ 6,510.17	4.52	$ 4,970.29	46.6	3.77	-10.5%	-11.2%	-33.9%	-2.2%	1.2%	-19.5%	-10.9%	**-12.4%**
**Congo**	AFR	0.42	$ 4,496.77	1.98	$ 5,870.54	1.03	$ 4,209.64	48.9	4.47	-13.0%	-14.2%	-45.4%	-3.1%	4.3%	-22.8%	-13.1%	**-15.3%**
**Côte d'Ivoire**	AFR	6.26	$ 5,101.84	6.58	$ 10,678.77	5.77	$ 7,892.54	39.35	3.72	-11.7%	-14.0%	-39.6%	-2.6%	10.6%	-20.4%	-12.1%	**-12.8%**
**Djibouti**	EMR	6.40	$ 5,556.25	6.00	$ 9,652.27	5.41	$ 9,025.18	41.6	2.01	-3.1%	-2.5%	-8.3%	0.1%	1.5%	-4.7%	-3.2%	**-2.9%**
**Egypt**	EMR	3.90	$ 11,940.25	5.98	$ 23,025.21	5.28	$ 18,304.59	31.7	4.36	-10.0%	-10.7%	-15.1%	-2.4%	-1.6%	-13.8%	-10.3%	**-9.1%**
**El Salvador**	AMR	2.49	$ 8,795.45	2.21	$ 11,230.33	1.89	$ 10,354.98	39	9.85	-3.5%	-3.4%	-4.6%	-0.3%	-0.9%	-4.8%	-4.0%	**-3.1%**
**Georgia**	EUR	4.86	$ 14,975.81	5.14	$ 25,857.63	4.48	$ 24,177.15	34.35	7.6	-3.1%	-2.7%	-4.3%	-0.3%	-0.1%	-3.9%	-3.5%	**-2.6%**
**Ghana**	AFR	6.66	$ 5,585.90	5.01	$ 9,628.29	4.89	$ 8,021.67	43.5	3.99	-7.0%	-7.3%	-20.5%	-1.1%	6.6%	-11.5%	-7.7%	**-6.9%**
**Guatemala**	AMR	3.54	$ 8,156.23	3.51	$ 12,088.17	3.34	$ 10,152.08	48.3	6.47	-7.6%	-7.7%	-23.8%	-0.9%	2.1%	-12.6%	-8.2%	**-8.4%**
**Honduras**	AMR	3.66	$ 5,721.95	3.85	$ 8,869.38	2.77	$ 6,790.51	49.1	9.04	-11.8%	-12.1%	-41.3%	1.3%	3.0%	-20.9%	-12.5%	**-13.5%**
**India**	SEAR	6.98	$ 6,708.12	7.33	$ 15,068.58	5.49	$ 11,242.61	35.1	2.96	-13.5%	-11.5%	-40.2%	0.3%	-7.1%	-21.8%	-13.5%	**-15.3%**
**Indonesia**	SEAR	5.43	$ 11,976.32	5.26	$ 21,262.70	4.60	$ 18,347.23	37.8	3.41	-6.3%	-5.8%	-18.9%	-1.1%	5.9%	-11.4%	-6.5%	**-6.3%**
**Jordan**	EMR	2.39	$ 10,103.11	2.89	$ 14,088.11	2.67	$ 12,729.53	33.7	7.47	-4.4%	-4.5%	-6.4%	-0.8%	-0.6%	-5.6%	-5.9%	**-4.0%**
**Kenya**	AFR	5.05	$ 4,918.07	5.86	$ 9,181.94	5.06	$ 7,237.66	40.8	4.29	-9.2%	-9.8%	-28.7%	-1.8%	1.2%	-17.0%	-9.8%	**-10.7%**
**Kiribati**	WPR	3.30	$ 2,130.46	1.90	$ 2,624.67	1.81	$ 2,314.98	27.8	11.64	-5.6%	-5.2%	-7.9%	-0.5%	4.8%	-8.5%	-7.5%	**-4.4%**
**Kyrgyzstan**	EUR	4.13	$ 5,298.56	3.51	$ 7,840.92	2.84	$ 6,202.94	29	5.26	-11.2%	-10.1%	-16.1%	-1.8%	5.2%	-14.1%	-11.2%	**-8.5%**
**Lao (PDR)**	WPR	7.15	$ 7,826.57	6.74	$ 16,566.77	4.71	$ 11,801.91	38.8	2.69	-15.6%	-15.4%	-56.3%	-2.8%	12.0%	-25.2%	-18.4%	**-17.4%**
**Lesotho**	AFR	1.90	$ 2,646.90	1.71	$ 3,348.64	0.83	$ 2,673.47	44.9	11.78	-8.7%	-9.2%	-9.5%	-1.6%	2.9%	-14.4%	-9.3%	**-7.1%**
**Mauritania**	AFR	4.29	$ 5,961.39	6.18	$ 11,534.01	4.03	$ 7,873.68	32.6	3.36	-14.9%	-18.3%	-69.3%	-10.1%	4.9%	-29.2%	-14.8%	**-21.7%**
**Micronesia**	WPR	0.72	$ 3,326.00	0.63	$ 3,578.95	0.44	$ 3,444.52	40.1	11.56	-1.9%	-1.5%	-2.3%	1.6%	0.2%	-2.8%	-1.4%	**-1.2%**
**Mongolia**	WPR	7.77	$ 12,048.93	5.19	$ 21,679.88	4.67	$ 17,631.69	32.5	4.94	-9.3%	-8.9%	-12.0%	-1.2%	2.2%	-14.5%	-9.8%	**-7.6%**
**Morocco**	EMR	3.49	$ 7,857.90	4.39	$ 12,740.77	2.79	$ 9,898.23	39.5	5.99	-11.1%	-10.2%	-16.8%	-2.7%	-1.8%	-18.0%	-11.2%	**-10.2%**
**Myanmar**	SEAR	6.61	$ 4,848.56	6.35	$ 9,567.18	0.44	$ 4,782.82	34.4	3.69	-31.8%	-33.8%	-261.0%	-12.2%	-0.1%	-62.5%	-24.7%	**-60.9%**
**Nicaragua**	AMR	3.39	$ 5,466.61	0.98	$ 6,020.54	2.47	$ 6,762.57	46.2	8.63	4.9%	4.6%	11.4%	-1.5%	-1.7%	7.5%	6.2%	**4.5%**

**Nigeria**	AFR	3.81	$ 5,135.49	2.62	$ 7,010.66	2.25	$ 5,510.27	35.5	3.38	-9.3%	-9.9%	-29.1%	-1.8%	3.1%	-15.5%	-9.9%	**-10.3%**
**Pakistan**	EMR	3.92	$ 5,000.79	4.55	$ 8,404.02	4.29	$ 6,962.17	30.4	2.95	-8.2%	-7.5%	-27.1%	-1.8%	4.2%	-13.4%	-8.6%	**-8.9%**
**Papua New Guinea**	WPR	5.44	$ 3,858.79	3.31	$ 5,589.17	2.21	$ 4,236.51	41.9	2.53	-12.6%	-12.3%	-42.1%	-1.9%	10.0%	-22.7%	-15.4%	**-13.9%**
**Philippines**	WPR	6.41	$ 8,982.57	6.46	$ 18,077.71	4.77	$ 13,343.13	40.7	5.11	-13.9%	-13.6%	-47.9%	-2.3%	2.8%	-22.2%	-13.8%	**-15.8%**
**Republic of Moldova**	EUR	4.31	$ 12,946.99	3.80	$ 19,074.53	4.11	$ 22,707.02	25.7	6.78	7.9%	6.4%	8.1%	-3.6%	3.5%	9.4%	10.2%	**6.0%**
**Sao Tome and Principe**	AFR	4.27	$ 4,009.05	4.36	$ 6,567.02	3.45	$ 4,825.22	40.7	4.91	-12.0%	-13.0%	-50.6%	-2.7%	4.0%	-20.3%	-12.3%	**-15.3%**
**Solomon Islands**	WPR	4.19	$ 2,378.38	2.83	$ 3,504.04	2.43	$ 2,651.65	37.1	4.43	-12.7%	-12.4%	-50.4%	-1.9%	10.1%	-22.9%	-15.5%	**-15.1%**
**Sri Lanka**	SEAR	5.25	$ 13,070.13	4.62	$ 21,707.53	3.30	$ 17,946.96	38.5	4.07	-8.2%	-9.1%	-10.1%	-1.5%	1.3%	-11.6%	-8.3%	**-6.8%**
**Sudan**	EMR	-0.85	$ 4,134.13	0.76	$ 4,622.06	4.74	$ 5,720.46	34.2	3.02	8.9%	7.3%	19.9%	0.4%	-5.1%	13.8%	14.4%	**8.5%**
**Swaziland**	AFR	2.62	$ 8,878.08	0.50	$ 9,387.43	1.58	$ 9,781.69	54.6	6.51	1.6%	1.5%	3.6%	0.1%	-1.6%	2.6%	1.9%	**1.4%**
**Tajikistan**	EUR	7.03	$ 3,399.97	4.14	$ 5,291.69	4.18	$ 4,707.91	34	8.18	-5.5%	-5.6%	-7.7%	4.2%	-0.3%	-6.8%	-5.1%	**-3.8%**
**Timor-Leste**	SEAR	3.08	$ 3,552.60	4.82	$ 5,351.26	1.82	$ 3,245.44	28.7	9.85	-22.3%	-26.9%	-108.8%	-6.9%	17.6%	-47.0%	-19.2%	**-30.5%**
**Tunisia**	EMR	1.94	$ 10,625.59	4.01	$ 16,625.97	1.42	$ 11,605.35	32.8	6.34	-16.0%	-15.2%	-24.8%	-4.6%	7.8%	-21.0%	-15.2%	**-12.7%**
**Ukraine**	EUR	0.49	$ 12,902.80	3.26	$ 18,206.38	3.11	$ 18,584.21	25.6	7.62	0.9%	1.1%	1.3%	0.1%	0.0%	1.2%	1.1%	**0.8%**
**Uzbekistan**	EUR	6.45	$ 7,419.93	6.00	$ 13,653.28	5.21	$ 11,564.24	34.8	6.75	-8.5%	-7.0%	-11.1%	-1.1%	3.8%	-9.8%	-10.1%	**-6.3%**
**Vanuatu**	WPR	2.72	$ 2,768.99	2.90	$ 3,916.94	1.94	$ 2,897.64	32.3	3.97	-13.8%	-17.6%	-56.3%	-2.2%	10.8%	-19.3%	-16.6%	**-16.4%**
**Viet Nam**	WPR	6.53	$ 10,131.71	6.50	$ 20,495.32	6.31	$ 18,661.48	36.8	4.68	-4.2%	-4.5%	-11.3%	-0.3%	3.6%	-7.0%	-3.7%	**-3.9%**
**Yemen**	EMR	-3.83	$ 1,960.36	5.02	$ 3,457.23	3.50	$ 2,410.84	36.7	4.25	-16.0%	-15.2%	-65.9%	-4.6%	7.9%	-28.7%	-15.2%	**-19.7%**
**Zambia**	AFR	4.89	$ 3,383.31	1.55	$ 4,153.38	0.96	$ 3,052.43	57.1	5.62	-12.0%	-13.0%	-40.6%	4.3%	10.8%	-22.7%	-12.3%	**-12.2%**

Notes: * Gini estimates. GDPpc and growth rates from IMF.

Source: own elaboration
