# Supplementary material for: Post-COVID-19 health inequalities: Estimates of the potential loss in the evolution of the health-related SDGs indicators
Source: PLoS One. 2024 Jul 24;19(7):e0305955. doi: 10.1371/journal.pone.0305955 (PMC11268624; doi:10.1371/journal.pone.0305955)
Supplement: S3 Table — Notes: * Gini estimates. GDPpc and growth rates from IMF. Source: own elaboration. (PDF) [file pone.0305955.s003.pdf]

S3 Table – Economic scenarios and mean estimated losses by health themes in 2030: upper middle-income countries

Country	WHO Region	Economic scenarios						Fixed covariates		Accumulated losses in the decade							
		Annual average growth rate (2010-2019)	GDP per capita (2019)	Growth rate Pre-COVID (2020-2030)	GDP per capita pre-Covid (2030)	Growth rate Post-COVID (2020-2030)	GDP per capita post-Covid (2030)	GINI index	Average Health expenditure (% GDP)	1. Maternal and reproductive health	2. Newborn and child health	3. Infectious diseases	4. Non-comunicable diseases	5. Injuries and violence	6. Environ-mental risks	7. Health systems and coverage	**Mean general loss**
**Albania**	EUR	2.60	$ 13,881.23	4.01	$ 21,661.14	3.20	$ 19,924.71	29.4	6.59	-1.9%	-3.6%	-11.6%	-1.3%	-5.5%	-3.3%	-2.5%	**-4.3%**
**Algeria**	AFR	2.68	$ 11,412.23	1.10	$ 13,360.90	0.34	$ 10,535.63	27.6	6.32	-4.4%	-9.8%	-37.3%	-4.1%	-16.9%	-8.8%	-6.7%	**-12.6%**
**Azerbaijan**	EUR	1.62	$ 14,501.14	2.34	$ 18,900.93	1.36	$ 15,494.80	26.6	4.61	-5.5%	-8.8%	-34.3%	-3.3%	-6.5%	-8.1%	-6.5%	**-10.4%**
**Belarus**	EUR	1.81	$ 19,267.04	-0.19	$ 18,753.22	1.06	$ 22,363.91	24.4	6.41	3.7%	7.7%	5.0%	2.4%	5.1%	6.7%	5.6%	**5.2%**
**Belize***	AMR	2.18	$ 6,929.95	1.74	$ 8,554.70	1.51	$ 6,951.27	49.8	6.92	-4.4%	-8.9%	-31.3%	-3.9%	-12.5%	-13.0%	-5.7%	**-11.4%**
**Bosnia and Herzegovina**	EUR	2.04	$ 14,993.05	2.90	$ 20,390.97	2.34	$ 19,895.47	33	9.84	-0.6%	-1.0%	-1.5%	-0.4%	-0.8%	-1.0%	-0.8%	**-0.8%**
**Botswana**	AFR	4.69	$ 16,386.87	4.14	$ 28,534.87	3.42	$ 20,341.00	53.3	6.19	-7.0%	-16.5%	-75.7%	-6.1%	-27.3%	-18.2%	-9.4%	**-22.9%**
**Brazil**	AMR	1.46	$ 14,827.29	2.27	$ 19,028.83	1.78	$ 17,259.89	50.9	10.31	-2.2%	-4.8%	-15.0%	-1.7%	-5.6%	-4.4%	-2.7%	**-5.2%**
**Bulgaria**	EUR	2.36	$ 23,346.14	2.85	$ 31,598.81	2.76	$ 32,725.74	40.5	8.52	0.9%	1.5%	5.6%	0.5%	2.2%	1.5%	1.1%	**1.9%**
**China**	WPR	7.67	$ 15,893.00	5.59	$ 29,267.64	5.15	$ 27,221.66	38.5	5.59	-2.1%	-3.3%	-10.9%	-1.0%	-3.9%	-3.0%	-2.0%	**-3.7%**
**Colombia**	AMR	3.72	$ 15,013.55	3.74	$ 22,302.89	2.92	$ 18,835.73	52.5	8.99	-3.5%	-7.5%	-28.8%	-3.1%	-10.0%	-9.3%	-4.6%	**-9.5%**
**Costa Rica**	AMR	3.76	$ 20,239.65	3.27	$ 28,913.11	2.64	$ 25,003.16	48.9	7.86	-3.0%	-6.5%	-23.9%	-2.6%	-8.5%	-6.5%	-4.0%	**-7.9%**
**Croatia**	EUR	1.03	$ 28,732.05	2.16	$ 36,202.15	2.75	$ 39,617.05	29.5	7.77	2.3%	4.1%	9.4%	1.3%	5.4%	3.7%	2.8%	**4.2%**
**Dominica***	AMR	0.76	$ 12,922.49	2.29	$ 15,269.66	2.84	$ 17,114.54	44	5.65	1.7%	4.8%	15.7%	3.4%	12.5%	5.3%	3.7%	**6.7%**
**Dominican Republic**	AMR	5.64	$ 19,090.48	5.02	$ 32,986.19	4.37	$ 28,541.57	39	4.94	-3.0%	-6.4%	-23.8%	-2.6%	-3.4%	-8.8%	-4.0%	**-7.4%**
**Ecuador**	AMR	2.83	$ 11,440.19	2.27	$ 14,770.86	1.84	$ 12,607.56	46.5	8.48	-3.3%	-7.1%	-26.5%	-2.9%	-3.8%	-8.7%	-4.4%	**-8.1%**
**Equatorial Guinea***	AFR	-3.36	$ 18,446.49	-3.53	$ 13,024.24	-2.16	$ 11,681.68	58.8	3.77	-2.0%	-4.6%	-22.0%	1.1%	-7.4%	-5.4%	-3.0%	**-6.2%**
**Fiji**	WPR	3.31	$ 13,611.33	3.18	$ 19,927.18	2.25	$ 16,331.39	30.7	3.75	-5.0%	-10.6%	-31.2%	-4.5%	-13.0%	-9.4%	-6.1%	**-11.4%**
**Gabon**	AFR	3.98	$ 15,608.06	4.36	$ 25,058.39	2.81	$ 19,432.42	38	3.43	-4.7%	-11.2%	-48.9%	-3.2%	1.6%	-13.3%	-6.8%	**-12.4%**
**Grenada***	AMR	2.85	$ 16,867.71	2.95	$ 23,843.18	2.16	$ 20,245.94	37	5.82	-3.7%	-7.7%	-32.6%	1.8%	-3.9%	-9.0%	-5.0%	**-8.6%**
**Guyana***	AMR	3.82	$ 13,052.24	14.53	$ 48,012.99	15.52	$ 56,006.54	46.7	5.51	3.0%	6.4%	16.9%	2.3%	7.7%	8.4%	5.0%	**7.1%**
**Iran**	EMR	0.84	$ 12,336.98	0.97	$ 13,466.41	2.16	$ 14,554.97	40.6	5.34	1.6%	3.5%	8.6%	1.3%	4.8%	2.8%	2.3%	**3.5%**
**Iraq**	EMR	6.11	$ 11,483.07	2.49	$ 14,522.60	2.37	$ 12,148.55	29.5	5.08	-3.9%	-7.4%	-40.6%	-3.4%	-12.8%	-7.0%	-5.0%	**-11.5%**
**Jamaica**	AMR	0.61	$ 10,544.60	2.00	$ 13,135.78	1.09	$ 11,692.23	46.9	6.61	-2.6%	-4.8%	-15.2%	-2.0%	-2.7%	-7.0%	-3.6%	**-5.4%**
**Kazakhstan**	EUR	4.47	$ 26,184.87	3.73	$ 39,410.72	3.22	$ 33,794.29	27.3	3.79	-3.6%	-6.7%	-24.4%	-2.5%	-10.5%	-5.9%	-4.6%	**-8.3%**
**Libya***	EMR	4.61	$ 14,007.05	0.00	$ 10,113.31	9.39	$ 17,334.29	55.9	4.82	10.1%	20.2%	34.6%	-3.8%	-3.5%	26.3%	19.3%	**14.8%**
**Malaysia**	WPR	5.35	$ 27,920.57	4.84	$ 47,175.25	4.08	$ 39,584.68	41.1	4.12	-4.4%	-7.6%	-26.4%	-4.0%	-10.2%	-7.6%	-5.2%	**-9.3%**
**Maldives**	SEAR	6.45	$ 27,893.66	5.52	$ 50,990.64	4.60	$ 37,036.98	30.3	11.35	-6.7%	-13.6%	-82.8%	-1.3%	-24.0%	-15.4%	-8.4%	**-21.7%**
**Marshall Islands**	WPR	2.30	$ 3,744.52	1.50	$ 4,254.33	1.30	$ 4,082.24	35.5	13.01	-0.4%	-1.8%	-7.0%	-1.2%	-4.5%	-2.1%	-1.3%	**-2.6%**
**Mauritius**	AFR	3.70	$ 22,852.14	3.99	$ 35,389.09	2.19	$ 28,509.53	36.8	6.66	-4.0%	-10.3%	-32.6%	-3.7%	-6.2%	-8.4%	-5.8%	**-10.1%**
**Mexico**	AMR	2.67	$ 19,927.66	2.21	$ 25,753.96	1.65	$ 22,328.21	45.4	6.24	-3.0%	-6.3%	-23.4%	-2.6%	-8.3%	-6.4%	-3.9%	**-7.7%**

**Montenegro**	EUR	2.87	$ 21,559.03	2.93	$ 29,312.21	1.96	$ 26,146.76	37.8	11.42	-2.6%	-4.7%	-16.8%	-1.8%	-7.7%	-3.3%	-3.4%	**-5.8%**
**Namibia**	AFR	3.17	$ 9,987.70	2.90	$ 13,991.21	1.60	$ 9,235.49	59.1	8.9	-7.9%	-17.8%	-107.1%	-7.8%	-35.5%	-23.0%	-10.9%	**-30.0%**
**Nauru**	WPR	9.81	$ 9,457.32	1.77	$ 11,063.99	0.70	$ 8,914.34	32.4	11.97	-2.2%	-11.1%	-48.8%	-2.9%	-	-11.9%	-6.4%	**-13.9%**
**North Macedonia**	EUR	2.56	$ 16,593.95	3.46	$ 24,109.27	2.98	$ 22,929.99	34.2	7.89	-1.1%	-2.1%	-9.2%	-0.6%	-1.6%	-2.0%	-1.7%	**-2.6%**
**Panama**	AMR	6.18	$ 31,637.97	5.50	$ 58,543.80	3.55	$ 41,160.17	50.9	9.66	-7.7%	-20.1%	-79.8%	-7.2%	-22.9%	-23.4%	-9.4%	**-24.4%**
**Paraguay**	AMR	4.31	$ 12,424.93	3.97	$ 19,645.00	3.29	$ 16,123.29	43.2	7.58	-4.2%	-8.9%	-29.3%	-3.7%	-11.8%	-9.0%	-5.4%	**-10.3%**
**Peru**	AMR	4.49	$ 12,783.13	3.80	$ 19,949.42	2.77	$ 15,900.97	42	6.3	-4.8%	-10.3%	-42.1%	-4.3%	-13.8%	-12.7%	-6.2%	**-13.4%**
**Romania**	EUR	3.10	$ 29,961.60	3.05	$ 41,376.75	3.34	$ 43,095.90	34.6	6.27	1.1%	2.0%	4.6%	0.6%	2.5%	1.6%	1.3%	**1.9%**
**Russian Federation**	EUR	2.10	$ 27,296.92	1.89	$ 33,231.59	1.67	$ 33,083.88	36	7.6	-0.1%	-0.2%	-0.8%	-0.1%	-0.3%	-0.2%	-0.1%	**-0.2%**
**Saint Lucia**	AMR	1.40	$ 15,452.24	1.87	$ 19,336.97	1.70	$ 17,174.26	51.2	6.74	-2.5%	-4.9%	-22.1%	1.3%	-6.8%	-7.2%	-3.3%	**-6.5%**
**St. Vincent/Grenadines***	AMR	0.76	$ 12,507.27	2.32	$ 16,408.65	2.49	$ 16,150.12	40	4.82	-0.3%	-0.7%	-2.6%	-0.2%	-0.9%	-0.9%	-0.5%	**-0.9%**
**Samoa**	WPR	1.72	$ 5,695.46	2.43	$ 7,313.49	1.57	$ 6,409.91	38.7	5.33	-2.8%	-5.6%	-25.8%	-2.9%	-7.5%	-5.7%	-4.1%	**-7.8%**
**Serbia**	EUR	1.93	$ 18,254.98	4.00	$ 27,822.44	3.89	$ 28,569.08	35	8.73	0.6%	1.1%	0.8%	0.4%	1.6%	1.0%	0.8%	**0.9%**
**South Africa**	AFR	1.73	$ 13,659.41	1.68	$ 15,229.54	1.02	$ 13,698.12	63.2	8.58	-1.9%	-4.2%	-15.9%	-1.7%	-7.0%	-4.0%	-2.9%	**-5.4%**
**Suriname***	AMR	1.62	$ 18,507.19	2.50	$ 21,772.58	-0.28	$ 16,324.22	57.9	6.77	-6.2%	-12.8%	-58.8%	-3.9%	-7.1%	-16.4%	-7.8%	**-16.1%**
**Thailand**	SEAR	3.65	$ 18,453.47	3.58	$ 27,398.37	2.56	$ 23,997.14	35	4.36	-2.7%	-5.3%	-21.7%	-2.4%	-8.7%	-6.1%	-3.6%	**-7.2%**
**Tonga**	WPR	2.29	$ 5,888.07	2.27	$ 7,737.58	1.81	$ 7,225.11	33.5	5.32	-1.4%	-2.9%	-12.1%	-0.4%	-4.2%	-2.9%	-2.1%	**-3.7%**
**Turkey**	EUR	5.86	$ 28,515.69	3.36	$ 41,325.78	3.69	$ 39,407.06	42	4.62	-1.1%	-2.0%	-8.7%	-0.7%	-3.1%	-2.0%	-1.4%	**-2.7%**
**Turkmenistan***	EUR	3.09	$ 14,249.69	5.86	$ 29,955.03	1.61	$ 15,732.98	51.1	5.68	-17.1%	-32.5%	-410.9%	-13.8%	0.2%	-28.9%	-19.4%	**-74.6%**
**Tuvalu**	WPR	4.06	$ 4,710.27	3.26	$ 5,971.06	3.42	$ 6,632.02	39.1	21.54	1.0%	4.7%	14.4%	1.3%	10.6%	5.3%	3.3%	**5.8%**

Notes: * Gini estimates. GDPpc and growth rates from IMF.

Source: own elaboration
