# Supplementary material for: Post-COVID-19 health inequalities: Estimates of the potential loss in the evolution of the health-related SDGs indicators
Source: PLoS One. 2024 Jul 24;19(7):e0305955. doi: 10.1371/journal.pone.0305955 (PMC11268624; doi:10.1371/journal.pone.0305955)
Supplement: S4 Table — Notes: * Gini estimates. GDPpc and growth rates from IMF. Source: own elaboration. (PDF) [file pone.0305955.s004.pdf]

S4 Table – Economic scenarios and mean estimated losses by health themes in 2030: high-income countries

		Economic scenarios						Fixed covariates		Accumulated losses in the decade							
**Country**	**WHO Region**	Annual average growth rate (2010-2019)	GDP per capita (2019)	Growth rate Pre-COVID (2020-2030)	GDP per capita pre-Covid (2030)	Growth rate Post-COVID (2020-2030)	GDP per capita post-Covid (2030)	GINI index	Average Health expenditure (% GDP)	1. Maternal and reproductive health	2. Newborn and child health	3. Infectious diseases	4. Non-comunicable diseases	5. Injuries and violence	6. Environ-mental risks	7. Health systems and coverage	**Total**
**Antigua and Barbuda***	AMR	2.09	$ 21,944.75	2.17	$ 28,004.37	1.46	$ 22,661.78	53	5.61	-10.0%	-9.8%	-23.4%	1.9%	17.6%	-19.6%	-4.8%	**-6.9%**
**Argentina**	AMR	1.38	$ 22,063.90	2.54	$ 29,044.20	1.33	$ 23,592.87	42.1	9.98	-9.8%	-7.6%	-18.7%	-1.0%	-3.4%	-16.5%	-4.2%	**-8.8%**
**Australia**	WPR	2.59	$ 50,542.12	2.61	$ 67,987.70	2.38	$ 61,661.87	34	10.65	-5.2%	-3.6%	-7.8%	-0.3%	-2.4%	-7.4%	-2.0%	**-4.1%**
**Austria**	EUR	1.55	$ 56,303.44	1.57	$ 66,762.30	1.61	$ 64,603.13	29.8	11.47	-1.7%	-1.5%	-3.4%	-0.2%	-0.7%	-2.1%	-0.8%	**-1.5%**
**Bahamas***	AMR	1.07	$ 37,269.26	1.45	$ 44,825.73	1.16	$ 38,530.96	53.3	7.59	-7.0%	-5.4%	-13.2%	-0.4%	-9.8%	-13.7%	-3.6%	**-7.6%**
**Bahrain***	EMR	3.44	$ 50,118.41	2.77	$ 66,437.55	2.29	$ 57,779.65	44.3	4.72	-8.0%	-5.0%	-16.3%	-1.7%	-9.7%	-8.7%	-3.2%	**-7.5%**
**Barbados***	AMR	-0.14	$ 15,444.60	1.70	$ 18,854.18	1.13	$ 16,760.34	47	7.2	-4.3%	-4.1%	-12.4%	-0.4%	-1.6%	-10.5%	-2.4%	**-5.1%**
**Belgium**	EUR	1.58	$ 52,078.71	1.35	$ 60,304.49	1.23	$ 57,740.55	26	11.06	-3.4%	-1.6%	-4.6%	-0.2%	-3.0%	-2.8%	-1.0%	**-2.4%**
**Brunei Darussalam***	WPR	0.51	$ 58,555.74	2.64	$ 79,402.65	2.06	$ 72,091.48	63.4	2.39	-5.2%	-4.5%	-8.7%	-0.3%	-6.8%	-9.1%	-2.2%	**-5.3%**
**Canada**	AMR	2.22	$ 49,392.36	1.71	$ 60,202.13	1.67	$ 55,097.00	33.05	12.94	-4.0%	-3.1%	-9.2%	-0.3%	-5.6%	-5.7%	-2.0%	**-4.3%**
**Chile**	AMR	3.30	$ 23,957.26	3.19	$ 34,996.46	2.34	$ 28,380.79	44.9	9.75	-9.9%	-7.7%	-18.9%	-1.0%	-13.8%	-14.2%	-4.6%	**-10.0%**
**Cyprus**	EUR	1.37	$ 40,522.28	2.57	$ 54,285.48	2.27	$ 46,982.61	31.7	8.09	-7.8%	-5.4%	-16.0%	-1.0%	-10.5%	-9.5%	-2.9%	**-7.6%**
**Czech Republic**	EUR	2.49	$ 41,223.23	2.55	$ 54,300.40	2.47	$ 53,001.84	26.2	9.24	-1.2%	-1.1%	-2.5%	-0.1%	-0.5%	-2.1%	-0.5%	**-1.2%**
**Denmark**	EUR	1.85	$ 57,409.97	1.58	$ 68,348.41	1.75	$ 67,972.52	27.5	10.53	-0.3%	-0.2%	-0.4%	0.0%	-0.1%	-0.3%	-0.1%	**-0.2%**
**Estonia**	EUR	3.64	$ 36,488.52	2.81	$ 49,397.97	3.28	$ 51,883.39	30.7	7.75	2.4%	2.1%	4.1%	0.2%	3.3%	2.9%	1.0%	**2.3%**
**Finland**	EUR	1.21	$ 48,682.99	1.37	$ 56,653.65	1.24	$ 55,509.35	27.1	9.61	-1.0%	-0.7%	-2.1%	-0.1%	-0.4%	-1.2%	-0.4%	**-0.9%**
**France**	EUR	1.42	$ 47,832.71	1.40	$ 55,555.44	1.26	$ 53,554.32	30.7	12.21	-1.9%	-1.3%	-3.8%	-0.2%	-0.8%	-2.3%	-0.8%	**-1.6%**
**Germany**	EUR	1.97	$ 54,234.43	1.22	$ 61,777.95	1.17	$ 61,217.02	31.6	12.82	-0.5%	-0.3%	-0.8%	0.0%	-0.6%	-0.6%	-0.2%	**-0.4%**
**Greece**	EUR	-2.10	$ 29,662.12	1.15	$ 33,638.04	1.42	$ 35,276.75	33.6	9.51	2.4%	1.6%	4.0%	0.2%	3.2%	2.8%	1.0%	**2.2%**
**Hungary**	EUR	2.81	$ 32,952.69	2.42	$ 42,819.31	2.76	$ 44,352.88	29.7	7.25	1.8%	1.2%	3.5%	0.2%	2.4%	2.2%	0.7%	**1.7%**
**Iceland**	EUR	2.86	$ 57,989.93	1.94	$ 72,101.64	1.94	$ 65,987.35	26.7	9.56	-4.7%	-3.3%	-8.0%	-0.5%	-6.3%	-5.7%	-2.0%	**-4.4%**
**Ireland**	EUR	6.29	$ 86,729.41	2.82	$119,294.80	4.23	$127,591.00	29.2	7.1	3.3%	2.9%	5.6%	0.3%	1.3%	4.1%	1.6%	**2.7%**
**Israel**	EUR	4.18	$ 40,710.86	3.03	$ 56,564.05	3.18	$ 50,575.40	38.9	8.32	-8.9%	-4.2%	-12.2%	-0.7%	-8.0%	-6.2%	-2.3%	**-6.1%**
**Italy**	EUR	0.26	$ 42,808.50	0.65	$ 45,345.43	0.88	$ 47,135.41	35.2	9.63	1.9%	1.3%	3.3%	0.2%	2.6%	2.4%	0.8%	**1.8%**
**Japan**	WPR	1.24	$ 41,819.77	0.50	$ 44,452.23	0.58	$ 45,826.45	32.5	10.9	1.6%	1.1%	2.6%	0.0%	2.2%	1.9%	0.6%	**1.4%**
**Kuwait***	EMR	1.62	$ 43,993.24	2.79	$ 61,537.20	1.61	$ 48,399.48	47.1	6.31	-14.4%	-9.1%	-29.7%	-3.7%	-18.9%	-15.6%	-5.4%	**-13.8%**
**Latvia**	EUR	2.54	$ 30,774.61	2.98	$ 42,521.11	2.89	$ 42,786.65	35.7	7.45	0.3%	0.2%	0.6%	0.0%	0.4%	0.4%	0.1%	**0.3%**
**Lithuania**	EUR	3.57	$ 37,133.51	2.35	$ 47,392.87	2.62	$ 49,294.09	36	7.54	2.0%	1.4%	4.0%	0.2%	2.7%	2.4%	0.9%	**1.9%**
**Luxembourg**	EUR	3.11	$115,602.31	2.61	$156,931.90	2.61	$134,313.30	33.4	5.77	-12.6%	-5.9%	-17.4%	-1.1%	-11.3%	-10.1%	-3.1%	**-8.8%**
**Malta**	EUR	5.95	$ 47,051.93	3.38	$ 67,795.32	3.07	$ 59,677.67	31.4	10.84	-6.8%	-6.0%	-14.0%	-0.8%	-9.2%	-8.1%	-2.6%	**-6.8%**
**Netherlands**	EUR	1.45	$ 57,258.26	1.53	$ 67,901.59	1.52	$ 65,815.21	26	11.14	-2.4%	-1.1%	-2.7%	-0.2%	-2.2%	-2.0%	-0.7%	**-1.6%**

**New Zealand***	WPR	2.90	$ 41,916.87	2.53	$ 56,144.49	2.17	$ 48,136.82	31.7	10.03	-8.4%	-5.8%	-12.6%	-0.6%	-4.0%	-10.1%	-3.5%	**-6.4%**
**Norway**	EUR	1.47	$ 63,230.51	1.74	$ 77,723.87	1.74	$ 73,323.26	27.6	11.42	-3.0%	-2.1%	-5.2%	-0.3%	-1.3%	-3.7%	-1.3%	**-2.4%**
**Oman***	EMR	3.01	$ 29,410.21	1.79	$ 36,158.21	2.50	$ 33,341.31	44.3	5.33	-4.5%	-2.9%	-9.2%	-0.9%	-6.0%	-5.6%	-1.8%	**-4.4%**
**Palau***	WPR	1.73	$ 15,351.06	2.00	$ 19,291.04	1.78	$ 16,810.82	39	18.39	-5.6%	-5.2%	-12.7%	-5.7%	10.2%	-5.1%	-3.2%	**-3.9%**
**Poland**	EUR	3.66	$ 33,284.21	2.56	$ 43,661.85	2.86	$ 45,809.26	30.3	6.49	2.4%	1.7%	4.8%	0.2%	3.3%	2.9%	1.0%	**2.3%**
**Portugal**	EUR	0.87	$ 34,989.23	1.51	$ 40,956.82	1.56	$ 41,709.90	34.7	10.55	0.9%	0.6%	1.6%	0.1%	0.4%	1.1%	0.4%	**0.7%**
**Qatar***	EMR	5.30	$ 91,101.14	2.73	$125,743.90	3.07	$117,304.10	46.2	4.18	-3.9%	-2.4%	-6.5%	-0.8%	-1.2%	-4.2%	-1.6%	**-2.9%**
**Saint Kitts and Nevis***	AMR	2.75	$ 26,718.42	2.78	$ 33,950.11	1.68	$ 29,347.63	40	5.37	0.0%	-6.5%	-15.6%	1.6%	12.5%	-10.2%	-3.4%	**-3.1%**
**San Marino***	EUR	-1.38	$ 61,649.02	0.53	$ 64,199.01	1.20	$ 68,360.14	34.5	8.69	4.5%	2.1%	4.6%	-	-	2.1%	1.5%	**3.0%**
**Saudi Arabia***	EMR	3.46	$ 47,031.29	2.41	$ 62,517.21	2.35	$ 52,950.51	45.6	6.0575	-9.6%	-6.1%	-16.4%	-2.4%	-12.7%	-12.7%	-3.8%	**-9.1%**
**Seychelles**	AFR	4.31	$ 28,036.58	3.67	$ 42,707.14	3.90	$ 39,507.92	32.1	6.39	-3.4%	-2.7%	-8.4%	-1.3%	-1.0%	-7.0%	-1.8%	**-3.6%**
**Singapore**	WPR	4.98	$ 98,411.58	2.24	$125,995.40	2.21	$121,345.20	37	6.05	-2.0%	-1.3%	-2.9%	0.0%	-2.7%	-2.4%	-0.8%	**-1.7%**
**Slovakia**	EUR	3.00	$ 32,760.06	2.59	$ 43,550.58	2.71	$ 43,452.60	24.6	7.23	-0.1%	-0.1%	-0.2%	0.0%	-0.2%	-0.1%	0.0%	**-0.1%**
**Slovenia**	EUR	1.92	$ 39,262.17	2.28	$ 50,476.21	2.80	$ 52,209.85	24	9.45	1.7%	1.2%	2.9%	0.2%	0.7%	2.0%	0.7%	**1.3%**
**South Korea**	WPR	3.33	$ 42,850.29	2.83	$ 58,204.75	2.42	$ 55,500.02	31.4	8.36	-2.5%	-1.7%	-4.2%	-0.1%	-1.1%	-3.0%	-1.0%	**-1.9%**
**Spain**	EUR	1.06	$ 40,875.30	1.64	$ 49,376.48	1.38	$ 46,087.21	34.9	10.71	-3.6%	-2.5%	-6.1%	-0.4%	-4.9%	-4.4%	-1.4%	**-3.3%**
**Sweden**	EUR	2.55	$ 53,402.35	1.94	$ 65,935.52	1.93	$ 61,287.73	28.9	11.38	-5.7%	-2.7%	-7.8%	-0.4%	-1.6%	-4.7%	-1.7%	**-3.5%**
**Switzerland**	EUR	2.02	$ 71,736.65	1.57	$ 85,426.30	1.49	$ 80,063.43	32.8	11.8	-5.1%	-2.4%	-5.0%	-0.4%	-1.4%	-4.2%	-1.5%	**-2.8%**
**Trinidad and Tobago***	AMR	-0.44	$ 25,827.83	1.73	$ 31,696.31	0.87	$ 27,355.31	53.3	7.31	-6.8%	-5.2%	-12.9%	0.7%	-2.2%	-13.3%	-3.2%	**-6.1%**
**United Arab Emirates**	EMR	3.75	$ 70,179.96	2.48	$ 82,180.77	2.27	$ 83,766.30	26	5.67	1.0%	0.6%	1.7%	0.0%	0.3%	1.4%	0.4%	**0.8%**
**United Kingdom**	EUR	1.83	$ 46,630.99	1.52	$ 55,247.17	1.32	$ 51,948.30	32.6	11.98	-4.8%	-2.2%	-6.5%	-0.4%	0.1%	-3.9%	-1.3%	**-2.7%**
**United States**	AMR	2.25	$ 62,417.55	1.66	$ 75,576.60	1.99	$ 75,401.22	39.7	18.82	-0.1%	-0.1%	-0.2%	0.0%	0.0%	-0.1%	-0.1%	**-0.1%**
**Uruguay**	AMR	2.89	$ 22,660.77	2.37	$ 29,448.02	1.71	$ 26,607.68	40.5	9.15	-4.6%	-3.5%	-8.7%	-0.4%	-1.4%	-8.9%	-2.1%	**-4.2%**

Notes: * Gini estimates. GDPpc and growth rates from IMF.

Source: own elaboration
