# Supplementary material for: Post-COVID-19 health inequalities: Estimates of the potential loss in the evolution of the health-related SDGs indicators
Source: PLoS One. 2024 Jul 24;19(7):e0305955. doi: 10.1371/journal.pone.0305955 (PMC11268624; doi:10.1371/journal.pone.0305955)
Supplement: S5 Table — Notes: Panel data estimations of Eq (1) by subsamples of the income strata. Significance level * p<0.1; ** p<0.05; *** p<0.01. Source: own estimations. (PDF) [file pone.0305955.s005.pdf]

S5 Table – Per capita GDP elasticity of the SDG indicator: by income strata

Indicator	Low-income	Lower-middle income	Upper-middle income	High-income
311	-0.9553***	-0.6700***	-0.5240***	-0.6469***
312	1.2884***	0.5027***	0.0756***	-0.0013
371	0.8708***	0.3235***	0.1108	-
372	-0.2662***	-0.2513	-0.0004	-0.6191***
223	-0.1897***	-0.1607***	-0.1784***	-
221	-0.5233***	-0.6709***	-0.6705***	-
222a	-0.6336**	-0.5923***	-0.2057	-
222b	-0.1571	0.0762	0.0304	-
321	-0.9076***	-0.9300***	-0.7729***	-0.8781***
322	-0.5744***	-0.7333***	-0.7580***	-0.8129***
3b1a	0.3715***	0.0893**	0.0434	-0.0014
3b1b	0.3026	0.1183	0.0704	-0.0139
3b1c	0.5100**	-0.047	0.6378***	-0.0306
331	-2.2294***	-0.0361	0.5591	-0.471
332	-0.7696***	-0.4735***	-0.3518**	-1.1742***
333	-1.0735***	-2.9735***	-2.2986***	-
334	-0.6661	-0.4277***	-0.6534	-0.9064
335	-0.6177	-0.6581	-3.6196**	-1.8182**
211	-0.9917***	-0.8753***	-0.7283***	-0.0937
341	0.4796***	0.7806***	0.4326**	0.3935**
342	-0.4546***	-0.0794	-0.1879	-0.6528***
352	0.5119**	0.5977**	0.0815	0.0685
3a1	-0.8321***	-0.5478***	-0.3452***	-0.4305***
1311	-1.2036	0.6281	-1.3279	0.9554
361	-0.0972*	0.1322**	0.0647	-0.7180***
1611	-	-0.5294	-0.5943***	-0.5651***
1623	-0.9341	-1.1350***	-	-
391	-0.2718*	-0.0367	-0.4143**	-0.9683***
392	-0.6007***	-1.2200***	-0.7435	-0.7056**
393	-0.4835***	-0.5630***	-0.4222***	-1.1047***
611	0.8434***	0.3348***	0.2094***	0.0797**
621a	0.9102***	0.5768***	0.4651**	0.2836***
621b	-1.1252***	-1.3774***	-1.0287***	-
712	0.3062**	0.7351***	0.2103***	-
1162	0.2358**	0.0854	-0.0539	-0.6697***
1a2	0.002	0.0051	0.1693***	0.2084**
381	0.6128***	0.4596***	0.3481***	0.2131***
382	0.2414	0.2114	-0.1589	-0.1758
3c1a	1.1848***	0.5275***	0.3063**	0.1757*
3c1b	1.0347***	0.4115***	0.3945***	0.4215***
3c1c	1.4254***	1.1323***	0.3581	0.3131
3c1d	1.8625***	1.0800***	0.2908	0.2645*
3d1	0.0037	0.2212***	0.1901**	0.0547

Notes: Panel data estimations of equation (1) by subsamples of the income strata. Significance level * p<0.1; ** p<0.05; *** p<0.01
Source: own elaboration
